# Cross-disorder risk gene *CACNA1C* differentially modulates susceptibility to psychiatric disorders during development and adulthood

**DOI:** 10.1038/mp.2017.133

**Published:** 2017-07-11

**Authors:** N Dedic, M L Pöhlmann, J S Richter, D Mehta, D Czamara, M W Metzger, J Dine, B T Bedenk, J Hartmann, K V Wagner, A Jurik, L M Almli, A Lori, S Moosmang, F Hofmann, C T Wotjak, G Rammes, M Eder, A Chen, K J Ressler, W Wurst, M V Schmidt, E B Binder, J M Deussing

**Affiliations:** 1 Molecular Neurogenetics, Department of Stress Neurobiology and Neurogenetics, Max Planck Institute of Psychiatry, Munich, Germany; 2 Queensland Brain Institute, University of Queensland, St. Lucia, QLD, Australia; 3 Department of Translational Research in Psychiatry, Max Planck Institute of Psychiatry, Munich, Germany; 4 Department of Psychiatry, Harvard Medical School and McLean Hospital, Belmont, MA, USA; 5 Institute of Pharmacology and Toxicology, Technische Universität München, Munich, Germany; 6 Department of Psychiatry and Behavioral Sciences, Emory University School of Medicine, Atlanta, GA, USA; 7 Clinic of Anaesthesiology, Klinikum Rechts der Isar, Technische Universität München, Munich, Germany; 8 The Ruhman Family Laboratory for Research on the Neurobiology of Stress, Department of Neurobiology, Weizmann Institute of Science, Rehovot, Israel; 9 Institute of Developmental Genetics, Helmholtz Zentrum München, German Research Center for Environmental Health, Neuherberg, Germany

## Abstract

Single-nucleotide polymorphisms (SNPs) in *CACNA1C*, the α1C subunit of the voltage-gated L-type calcium channel Ca_v_1.2, rank among the most consistent and replicable genetics findings in psychiatry and have been associated with schizophrenia, bipolar disorder and major depression. However, genetic variants of complex diseases often only confer a marginal increase in disease risk, which is additionally influenced by the environment. Here we show that embryonic deletion of *Cacna1c* in forebrain glutamatergic neurons promotes the manifestation of endophenotypes related to psychiatric disorders including cognitive decline, impaired synaptic plasticity, reduced sociability, hyperactivity and increased anxiety. Additional analyses revealed that depletion of *Cacna1c* during embryonic development also increases the susceptibility to chronic stress, which suggest that Ca_v_1.2 interacts with the environment to shape disease vulnerability. Remarkably, this was not observed when *Cacna1c* was deleted in glutamatergic neurons during adulthood, where the later deletion even improved cognitive flexibility, strengthened synaptic plasticity and induced stress resilience. In a parallel gene × environment design in humans, we additionally demonstrate that SNPs in *CACNA1C* significantly interact with adverse life events to alter the risk to develop symptoms of psychiatric disorders. Overall, our results further validate *Cacna1c* as a cross-disorder risk gene in mice and humans, and additionally suggest a differential role for Ca_v_1.2 during development and adulthood in shaping cognition, sociability, emotional behavior and stress susceptibility. This may prompt the consideration for pharmacological manipulation of Ca_v_1.2 in neuropsychiatric disorders with developmental and/or stress-related origins.

## Introduction

Major psychiatric disorders including schizophrenia (SCZ), bipolar disorder (BPD), major depressive disorder (MDD) and autism are moderately to highly heritable, and increasing evidence is suggesting a shared genetic etiology among them.^1, 2, 3, 4, 5, 6^ This might partially explain why many patients suffering from these illnesses display a substantial amount of overlapping symptoms.^7^ One of the most consistent and robust genetic findings from genome-wide association studies (GWAS) and meta-analyses of GWAS are associations of single-nucleotide polymorphisms (SNPs) in the α1 subunit (*CACNA1C*) of the voltage-gated L-type Ca^2+^ channel (LTCC) Ca_v_1.2 with SCZ and BPD, and to a lesser extent with MDD and autism.^1, 8, 9, 10, 11, 12, 13, 14, 15, 16^ In support, candidate analysis studies have clearly indicated a shared genetic risk for *CACNA1C* across these disorders.^1, 15, 17^ Further evidence comes from clinical studies, which have associated the primary disease-associated *CACNA1C* risk allele, rs1006737, with variations in human brain function and structure in patients, but also in healthy subjects.^18, 19, 20^ Hence, the available GWAS and clinical data establish CACNA1C as a possible shared susceptibility factor which influences disease vulnerability for BPD, SCZ and MDD across current diagnostic boundaries. This is of considerable interest, in view of the fact that LTCCs have a pivotal role in modulating neuronal excitability, synaptic plasticity and gene expression.^21, 22, 23^ However, the causality and mechanisms of how genetic alterations in CACNA1C affect the risk for an entire spectrum of psychiatric disorders remain largely unknown. Importantly, the associated *CACNA1C* variants by themselves only confer a marginal increase in disease risk. In addition to shared genetic risk factors, the environment represents an important contributor to the risk for many psychiatric disorders. In particular, adverse life events such as severe trauma and/or chronic stress represent strong risk factors not only for MDD but also for other psychiatric disorders including BPD and SCZ.^24, 25, 26^ However, no study to date has examined whether the established risk gene *CACNA1C* interacts with established environmental risk factors to shape disease outcome.

Functional LTCCs are hetero-oligomeric complexes consisting of multiple subunits: α1, β, α2, δ and/or γ. The voltage sensor, selectivity filter, ion-conduction pore and binding site for all available calcium channel blockers is encoded by the α1 subunit. The LTCC family consists of four distinct members, Ca_v_1.1–Ca_v_1.4, with mainly Ca_v_1.2 and Ca_v_1.3 shown to have a prominent role in the brain,^19, 22, 23^ and Ca_v_1.2 accounting for ~85% of the LTCCs.^27^ Pharmacological agents, such as the LTCC-targeting dihydropyridines, have frequently been applied to assess the function of LTCCs in the central nervous system (CNS). However, this approach is limited by the fact that all LTCC antagonists available to date are not completely selective for either Ca_v_1.2 or Ca_v_1.3, and that most studies investigated acute rather than long-term effects. More sophisticated genetic approaches using transgenic mice have helped to address the selective functional roles of different LTCCs.^19, 22, 23^ Constitutive deletion of *Cacna1c* was shown to result in embryonic lethality^28^ and conditional inactivation of Ca_v_1.2 in forebrain structures was repeatedly associated with impairments in cognitive function.^29, 30, 31, 32^ More recently, haploinsufficiency and forebrain-specific deletion of *Cacna1c* were also associated with anxiety-like behavior^33, 34^ and sleep disturbances,^35^ two core endophenotypes of MDD and BPD. In addition, *Cacna1c* was shown to mediate survival of young hippocampal neurons^36^ and brain-specific deletion of *Cacna1c* impairs discrete forms of hippocampal-dependent memory and neurogenesis within the dentate gyrus.^37^ However, the underlying neuronal circuits that modulate the effects of CACNA1C on synaptic plasticity and behavior remain largely unknown.

In this study we aimed to investigate how CACNA1C modulates the risk to psychiatric disorders in dependence of environmental, developmental and circuit specific factors, using both genetic mouse models and human data.

## Materials and methods

For a detailed description of the Materials and Methods, please refer to the Supplementary Information.

### Animals

Male mice were used for all experiments. Inactivation of *Cacna1c* from forebrain glutamatergic neurons during development was achieved by breeding *Cacna1c*^*lox*/lox^ mice^29^ to *Nex-Cre* mice,^38^ to obtain *Cav1.2-Dev*^*Glu-Ctrl*^ (*Cacna1c*^*lox/lox*^) and *Cav1.2-Dev*^*Glu-CKO*^ (*Cacna1c*^*lox/lox*^*:Nex-Cre*). Homozygous and heterozygous deletion of *Cacna1c* from forebrain excitatory projection neurons in adulthood was achieved by breeding *Cacna1c*^*lox/lox*^ mice to transgenic *Camk2α-CreER*^*T*2^ mice^39^ to obtain *Cav1.2-Ad*^*Glu-Ctrl*^ (*Cacna1c*^*lox/lox*^) and *Cav1.2-Ad*^*Glu-CKO*^ (*Cacna1*^*lox/lox*^*:Camk2α-CreER*^*T2*^), as well as *Cav1.2-Ad*^*Glu-Ctrl*^ (*Cacna1c*^*+/lox*^) and *Cav1.2-Ad*^*Glu-Het*^ (*Cacna1*^*+/lox*^*:Camk2α-CreER*^*T2*^), respectively. *Cacna1c* inactivation in *Cav1.2-Ad*^*Glu-CKO*^ and *Cav1.2-Ad*^*Glu-Het*^ mice was induced via 2 weeks of tamoxifen-containing food administration initiated during postnatal weeks 11–13. Both, control (Ctrl) and conditional knockout (CKO; CKO^het^) mice received the identical tamoxifen diet. Behavioral experiments were started following an additional two-week washout period in which *Cav1.2-Ad*^*Glu-CKO*^ and *Cav1.2-Ad*^*Glu-Het*^ mice received regular chow. Adult male CNS-specific *Cacna1c* knockout mice and Ctrl littermates were obtained by initially breeding *Cacna1c*^*lox*/lox^ CKO with Nestin-Cre mice.^40^ Subsequently *Cacna1c*^*lox*/lox^ mice were bred to *Cacna1c*^*−/+*^*:Nestin-Cre* mice to obtain *Cav1.2*^*CNS-Ctrl*^ (*Cacna1c*^*+/lox*^*:Nestin-Cre)* and *Cav1.2*^*CNS-CKO*^ (*Cacna1c*^*−/lox*^:*Nestin-Cre*). Heterozygous *Cav1*.2 mice and their Ctrl littermates were obtained from the same breedings; *Cav1.2*^*Ctrl*^ (*Cacna1c*^*+/lox*^) and *Cav1.2*^*Het*^ (*Cacna1c*^*−/lox*^). All animals were kept under standard laboratory conditions and were maintained on a 12 h light–dark cycle (lights on from 0700 to 1900 h), with food and water provided *ad libitum*. All experiments were conducted in accordance with the Guide for the Care and Use of Laboratory Animals of the Government of Upper Bavaria, Germany.

### Chronic social defeat stress paradigm

The chronic social defeat stress (CSDS) paradigm is commonly applied to induce anxiety- and depression-related endophenotypes in mice, and was performed as previously described.^41, 42, 43^ See also Supplementary Information.

### Behavioral testing and study design

Locomotion and sociability were investigated with the open field (OF) test and the three chamber apparatus respectively, as previously described.^41, 44^ Anxiety-related behaviour was assessed in the light/dark box test as previously described.^45^ Spatial memory learning and object recognition were investigated with the water cross maze (WCM)^46^ and spatial object recognition task. The forced swim test (FST) was used to assess passive vs active stress-coping behavior (behavioral despair) and corticosterone levels in response to an acute stressor as previously described.^41^ For details, refer to online Supplementary Information.

For the basal behavioral characterization of *Cav1.2-Dev*^*Glu-CKO*^ (Figure 2), *Cav1.2-Ad*^*Glu-CKO*^ (Figure 2) and *Cav1.2*^*CNS-CKO*^ mice (Supplementary Figure S7), one batch of animals was used for each line with the following order of tests (OF, sociability, dark/light box test, FST and corticosterone assessment). A second and third batch of animals was used for the WCM test and long-term potentiation (LTP) recordings, respectively. In addition, a fourth batch of *Cav1.2-Ad*^*Glu-CKO*^ mice (Figure 4) was used for the CSDS paradigm with the following test order (sociability, OF, object recognition test, dark/light box, FST and subsequent corticosterone, adrenal gland and thymus weight assessment). The same applies to *Cav1.2-Ad*^*Glu-Het*^ mice (Supplementary Figure S9). For the CSDS experiment in *Cav1.2*^*Het*^ mice (Figure 3), two separate batches of animals were used. For batch 1, the OF and dark/light box test were performed. For batch 2, the following tests were performed in this order: sociability, object recognition test, FST and subsequent corticosterone, adrenal gland and thymus weight assessment.

### Electrophysiology

The influence of *Cacna1c* deficiency on hippocampal LTP was conducted as previously described.^47^ For details, please refer to online Supplementary Information.

### Single and double *in situ* hybridization

Single and double *in situ* hybridization (ISH) was performed as previously described.^48^ The *Cacna1c* probe was designed to target the loxP flanked exons 14 and 15 (nucleotides 2307–2427 of GenBank accession number NM_009781). In addition, the following riboprobes were used: *Gad67*: 984–1940 of NM_008077; *Gad65*: 753–1600 of NM_008078; *Vglut1 (Slc17a7)*: 1716–2332 of NM_010484; *Vglut2* (Slc17a6): 2427–3006 of NM_080853.3; and *LacZ*: 2649–3281 of X65335. Images were analyzed with ImageJ (http://rsweb.nih.gov/ij/). Three to four independent experiments were performed for all ISHs and double ISHs.

### Study design (gene × environment interaction in humans)

Participants in this study belonged to a larger cohort (Grady Trauma Project) investigating the role of genetic and environmental factors in predicting outcomes to stressful life events.^49, 50, 51^ Phenotypes and genotypes were available for a total of 4,023 individuals, predominantly African Americans belonging to a highly traumatized, urban population of low socioeconomic status. All procedures were approved by the institutional review boards of Emory University School of Medicine and Grady Memorial Hospital. Please see Supplementary Methods for further details and statistics.

### Statistical analysis

Statistical analyses were performed using the commercially available software SPSS v16.0 (SPSS, Chicago, IL, USA) and GraphPad Prism v5.0 (GraphPad Software, La Jolla, CA, USA). The sample size was chosen such that with a type 1 error of 0.05 and a type 2 error of 0.2 the effect size should be at least 1.2-fold of the pooled s.d. All results are presented as mean±s.e.m. Behavioral phenotypic differences between two genotypes were evaluated with Student’s *t*-test (two-tailed). If data were not normally distributed, the non-parametric Mann–Whitney test was used. Time-dependent measures were assessed with multi-factorial analysis of variance (ANOVA) with repeated measures. For CSDS experiments, the effects of genotype and condition on all other behavioral and neuroendocrine parameters were assessed by two factorial ANOVA (two-way ANOVA). Whenever significant main or interaction effects were found by the ANOVAs, Bonferroni *post hoc* tests were carried out to locate simple effects. Statistical significance was defined as *P*<0.05. All data were tested for outlieres using the Grubb’ test. Homogeneity of variance was tested using Bartlett’s test. Animals were allocated to the experimental groups in a semi-randomized manner and data analysis was performed blinded to the group allocation.

## Results

### Forebrain *Cacna1c* is predominately expressed in glutamatergic neurons

**Figure 1:**
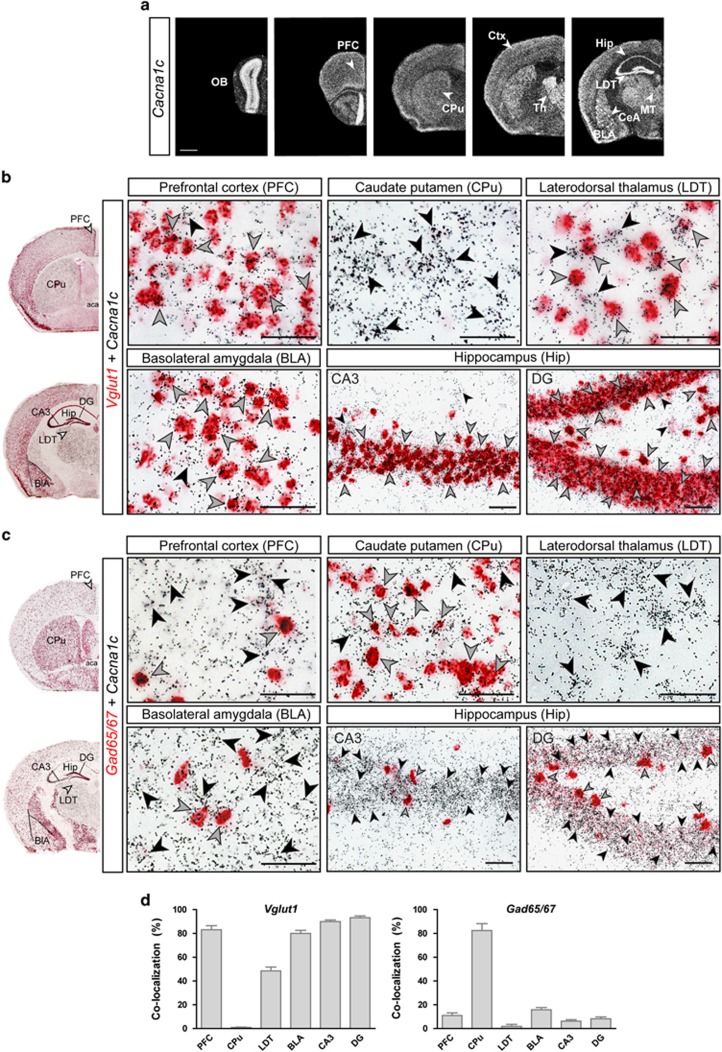
*Cacna1c* is predominantly expressed in forebrain glutamatergic neurons. (**a**) *Cacna1c* mRNA expression in the mouse brain (C57BL/6J mouse strain), determined by *in situ* hybridization (ISH). (**b** and **c**) Co-localization of *Cacna1c* with glutamatergic (*Vglut1*) and GABAergic (*Gad65/67*) markers with double ISH (DISH). Black arrowheads indicate cells only expressing *Cacna1c* (silver grains). Gray arrowheads indicate cells co-expressing *Cacna1c* and the respective neurotransmitter marker (red staining). (**d**) Quantifications of *Cacna1c* co-expression with *Vglut1* and *Gad65/67* (*n*=3, 2–3 sections per mouse). Scale bar=ISH, 1 mm; DISH, 50 μm.

*Cacna1c* is expressed throughout the mouse brain, including key limbic regions relevant for emotion and cognition such as the prefrontal cortex (Ctx), hippocampus (Hip) and amygdala (Figure 1a). Double ISH against *Cacna1c* and markers of excitatory glutamatergic (*Vglut1* and *Vglut2*), as well as inhibitory GABAergic neurons (*Gad65/Gad67*), revealed a predominant expression of *Cacna1c* in glutamatergic *Vglut1*-positive neurons throughout the Ctx, Hip and basolateral amygdala. The prominent *Cacna1c* expression in the thalamus was largely restricted to glutamatergic *Vglut2*-positive neurons, although a number of *Cacna1c*-containing neurons within the latero-dorsal thalamus also co-expressed *Vglut1* (Figures 1b and d and Supplementary Figure S1e,f). Minimal to no co-expression with *Vglut1* was detected in the caudate putamen, central nucleus of the amygdala, bed nucleus of the stria terminalis or granule cell layer of the olfactory bulb, confirming the abundance of GABAergic markers in these regions. Accordingly, *Cacna1c* expression in the olfactory bulb, CPu, bed nucleus of the stria terminalis and central nucleus of the amygdala was mainly restricted to *Gad65/67*-positive GABAergic neurons. In addition, *Cacna1c* was also detected in scattered *Gad65/67*-positive neurons of the Hip and Ctx (Figures 1c and d and Supplementary Figure S1c,d).

### Deletion of *Cacna1c* from forebrain glutamatergic neurons during development and adulthood induces differential effects on anxiety and opposing effects on cognition

**Figure 2:**
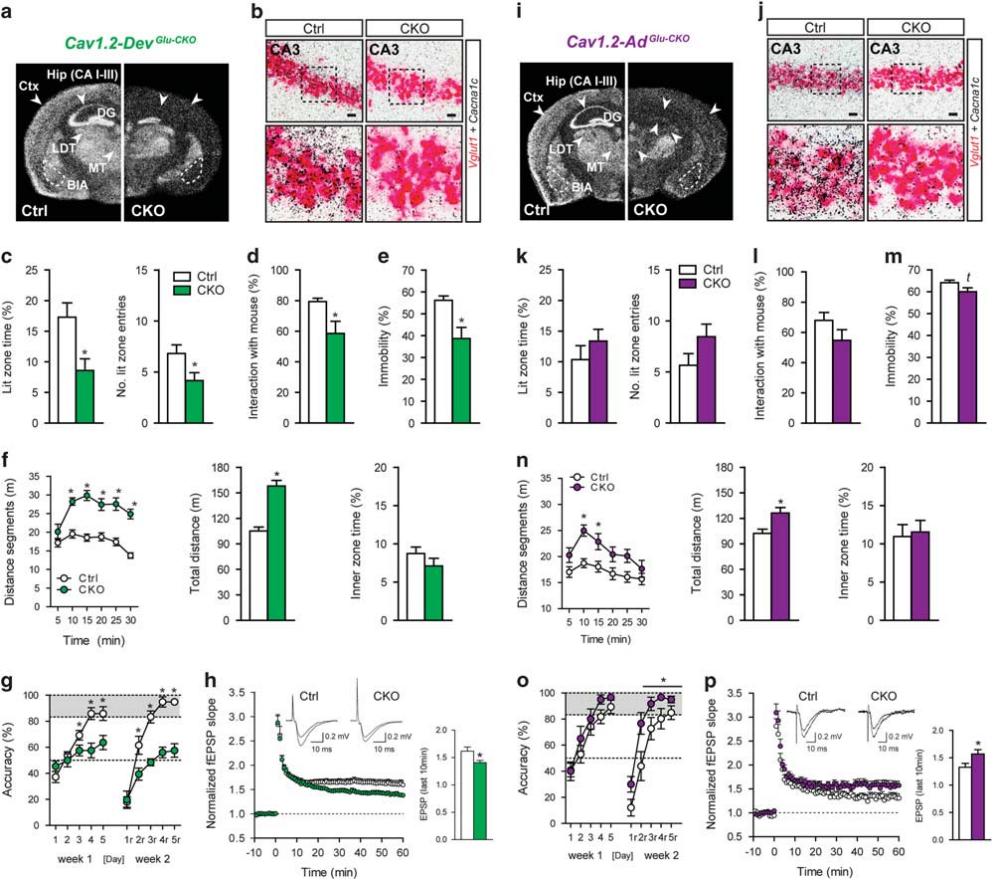
Absence of *Cacna1c* in forebrain glutamatergic neurons during development, but not adulthood, promotes aversive behavioral and cognitive deficits. (**a**) *Cacna1c* expression pattern determined by *in situ* hybridization (ISH) following developmental deletion of *Cacna1c* (E11.5) in forebrain glutamatergic neurons (*Cav1.2-Dev*^*Glu-CKO*^). (**b**) Double ISH (DISH) in *Cav1.2-Dev*^*Glu-CKO*^ mice demonstrates absence of *Cacna1c* mRNA expression (silver grains) in *Vglut1*-positive neurons (red staining) in the hippocampal CA3 region (conditional knockout (CKO)=3.6±0.74% double-positive neurons normalized to control (Ctrl); one-way analysis of variance (ANOVA): F_1,11_=365.4, *P*<0.0001, *n*=4, 1–2 sections per mouse). Enlarged images of boxed areas are shown in the bottom panel. (**c**) Dark/light box test (lit time: *t*_26_=2.7, *P*<0.05; No. lit entries: *t*_26_=2.2, *P*<0.05; *n*=16 Ctrl, 12 CKO). (**d**) Sociability test (*t*_26_=2.9, *P*<0.05; *n*=16 Ctrl, 12 CKO). (**e**) Forced swim test (FST; *t*_26_=3.6, *P*<0.005; *n*=16 Ctrl, 12 CKO). (**f**) Distance travelled an inner zone time in the open field (OF) test (distance segments: repeated measures (RM)-ANOVA:time × genotype: F_5,130_=5.7, *P*<0.0001; genotype: F_(1,130)_=45.9, *P*<0.001; total distance: *t*_26_=6.8, *P*<0.0001; *n*=16 Ctrl, 12 CKO). (**g**) Water cross-maze test (RM-ANOVA: genotype: F_1,88_=7.5, *P*<0.05; time × genotype: F_4,88_=7.3, *P*<0.0001; *n*=13 Ctrl, 11 CKO). (**h**) Schaffer collateral/CA1-long-term potentiation (LTP) in *Cav1.2-Dev*^*Glu-CKO*^mice (fEPSP last 10 min: *t*_24_=2.36, *P*<0.05; *n*=14 slices from 4 Ctrl mice and 12 slices from 4 CKO mice (**i**) *Cacna1c* expression pattern determined by ISH following adult-specific deletion (postnatal week 11–13) of *Cacna1c* in forebrain glutamatergic neurons (*Cav1.2-Ad*^*Glu-CKO*^). (**j**) DISHs in *Cav1.2-Ad*^*Glu-CKO*^ mice demonstrated the absence of *Cacna1c* mRNA expression in *Vglut1*-positive neurons in the hippocampal CA3 region (CKO=4.5±1.6% double-positive neurons normalized to Ctrl; one-way ANOVA: F_1,11_=365.4, *P*<0.0001, *n*=4, 1–2 sections per mouse). Enlarged images of boxed areas are shown in the bottom panel. (**k**–**m**) Dark/light box, sociability and FST (*t*=trend, *P*=0.09, *n*=17 Ctrl, 11 CKO) in *Cav1.2-Ad*^*Glu-CKO*^ mice. (**n**) OF test (distance segments: RM-ANOVA: genotype, F_1,130_=9.06, *P*<0.05; total distance: *t*_26_=3.01, *P*<0.05; *n*=17 Ctrl, 11 CKO) in *Cav1.2-Ad*^*Glu-CKO*^ mice. (**o**) Water cross-maze test (RM-ANOVA: genotype, F_1,80_=4.36, *P*<0.05; *n*=11 Ctrl, 10 CKO) in *Cav1.2-Ad*^*Glu-CKO*^ mice. (**p**) Schaffer collateral/CA1-LTP in *Cav1.2-Ad*^*Glu-CKO*^ mice (fEPSP last 10 min: *t*_27_=2.06, *P*<0.05; *n*=13 slices from 6 Ctrl mice and 16 slices from 7 CKO mice). Student’s *t*-test for simple comparisons, *P<0.05. Data are means±s.e.m.

In view of the results described above, we set out to genetically dissect the role of *Cacna1c* in glutamatergic neurons. For this, we crossed floxed *Cacna1c* mice to *Nex-Cre* mice,^38^ in which Cre-mediated recombination is initiated during embryonic development (E11.5) in forebrain glutamatergic neurons. As observed from the ISHs and double ISHs, *Cacna1c* ablation in forebrain glutamatergic neurons (*Cav1.2-Dev*^*Glu-CKO*^) was largely restricted to *Vglut1*-expressing cells, including the Ctx, CA1/2/3 of the Hip, and lateral and basolateral amygdala (Figures 2a and b, and Supplementary Figures S2). Next, we assessed behavioral endophenotypes known to be altered in animal models of MDD, BPD and SCZ, including locomotion/exploration in the OF test, anxiety in the dark/light box test, immobility in the FST, social behavior in the three-chamber test (sociability test), as well as spatial learning and memory in the WCM task. Similar to humans, deficits in cognition and social behavior are recapitulated by mouse models of numerous psychiatric disorders including SCZ, BPD and MDD.^52, 53, 54^ Hyperactivity in the OF and FST are often associated with mania in mouse models of BPD,^53, 55^ whereas enhanced anxiety represents a core endophenotype of MDD and the depressive phase of BPD in rodents.^52^ However, changes in locomotor activity are also commonly reported in animal models of SCZ and MDD.^52, 54^ Compared with their respective littermate Ctrls, *Cav1.2-Dev*^*Glu-CKO*^ mice exhibited increased anxiety in the dark–light box test and decreased preference for the social counterpart in the sociability test (Figures 2c and d). In the FST, *Cav1.2-Dev*^*Glu-CKO*^ mice exhibited increased active stress-coping behavior (reduced behavioral despair), evident by decreased immobility (Figure 2e). In addition, *Cav1.2-Dev*^*Glu-CKO*^ mice displayed a pronounced hyperlocomotion in the OF test, which was not observed during the initial 5 min where novelty-induced anxiety is most prominent (Figure 2f). The OF was performed under low light conditions (15 lux) in order to minimize anxiety effects on locomotion, which likely explains the lack of effects on inner zone time (Figure 2f). Hip-dependent spatial learning and memory performance was investigated in the WCM.^46^ Compared with the regular Morris water-maze test, the simplicity of the WCM leads to short trial durations and therefore reduces the stress load compared to Morris water-maze training. In addition, using accuracy rather than speed as the main readout, allows for hippocampus-dependent strategies to be assessed from the first training day on. *Cav1.2-Dev*^*Glu-CKO*^ mice displayed accuracy levels that barely surpassed the chance level of 50%, both during learning and relearning (Figure 2g), suggesting drastically impaired cognitive performance. In view of this strong effect on cognition, we additionally assessed hippocampal LTP at the Schaffer collateral-CA1 synapses. One hour after a 100 Hz tetanus stimulation, *Cav1.2-Dev*^*Glu-CKO*^ showed a significant decrease in LTP compared with Ctrl mice (Figure 2h).

Next we addressed whether the underlying neurobiological changes responsible for the behavioral alterations in *Cav1.2-Dev*^*Glu-CKO*^ mice occur during development or adulthood. Floxed *Cacna1c* mice were bred to inducible *Camk2α-CreER*^*T2*^ animals^39^ with the aim of deleting *Cacna1c* in forebrain glutamatergic neurons (*Cav1.2-Ad*^*Glu-CKO*^) during adulthood (postnatal weeks 11–13). We deliberately chose the *Camk2α-CreER*^*T2*^ line because of the strongly overlapping expression pattern with the *Nex-Cre* line (Figures 2a and i, and Supplementary Figures S2 and S5). Ca_v_1.2 was inactivated upon tamoxifen administration in forebrain excitatory projection neurons, which predominantly include glutamatergic pyramidal neurons of the Ctx, Hip and basolateral amygdala.^39^ As expected, the mRNA deletion pattern strongly resembled that of *Cav1.2-Dev*^*Glu-CKO*^ mice, with loss of *Cacna1c* expression in *Vglut1*-positive neurons of the lateral and basolateral amygdala, as well as the entire cerebral Ctx and Hip (Figures 2i and j, and Supplementary Figures S3 and S4). Along these lines, remaining hippocampal protein levels did not significantly differ between *Cav1.2-Dev*^*Glu-CKO*^ and *Cav1.2-Ad*^*Glu-CKO*^(Supplementary Figure S6a,b). In addition, deletion of *Cacna1c* mRNA expression in *Cav1.2-Dev*^*Glu-CKO*^ mice was also observed throughout the DG and within a few neurons of the latero-dorsal thalamus and medial parts of the thalamus, central nucleus of the amygdala and the geniculate nucleus (Supplementary Figures S2). In the OF, *Cav1.2-Ad*^*Glu-CKO*^ mice displayed enhanced locomotion (Figure 2n), although this was not as strongly pronounced as in *Cav1.2-Dev*^*Glu-CKO*^ mice. However, immobility in the FST and sociability were not significantly altered in *Cav1.2-Dev*^*Glu-CKO*^ mice (Figures 2l and m). Surprisingly, *Cav1.2-Ad*^*Glu-CKO*^ mice displayed a marginal increase in time spent in the lit zone and number of entries in the dark/light box test (Figure 2k), and even demonstrated enhanced cognitive flexibility during the relearning trial of the WCM (Figure 2o). Considering that deletion of forebrain *Cacna1c* was reported to impair long-term memory,^32^ we re-exposed the animals to the WCM 30 days after relearning, but did not observe any differences between *Cav1.2-Ad*^*Glu-CKO*^ and Ctrl mice (Supplementary Figure S6g). In view of our results that developmental inactivation of *Cacna1c* from glutamatergic neurons decreases NMDA receptor (NMDAR)-dependent synaptic plasticity and impairs spatial memory, we additionally assessed hippocampal LTP in *Cav1.2-Ad*^*Glu-CKO*^ mice. Intriguingly, 1 h after a 100 Hz tetanus stimulation, *Cav1.2-Ad*^*Glu-CKO*^ showed increased LTP compared with Ctrl mice (Figure 2p). Importantly, we observed that CNS-specific *Cacna1c* deletion (*Cav1.2*^*CNS-CKO*^ mice), which is initiated at E8,^56^ results in similar behavioral alterations compared with *Cav1.2-Dev*^*Glu-CKO*^ mice (Supplementary Figure S7). This further supports partially opposing roles for developmental and adult *Cacna1c* in emotion and cognition, and argues against the possibility that differences between *Cav1.2-Dev*^*Glu-CKO*^ and *Cav1.2-Ad*^*Glu-CKO*^ mice are due to the slight discrepancies in recombination patterns between the *Nex-Cre* and *Camk2a-CreERT2*. In addition, the observed behavioral changes were not caused by alterations in hypothalamic-pituitary-adrenal axis function in neither of the mouse lines (Supplementary Figures S6c,d).

### *Cacna1c* differentially modulates susceptibility to CSDS during development and adulthood

**Figure 3:**
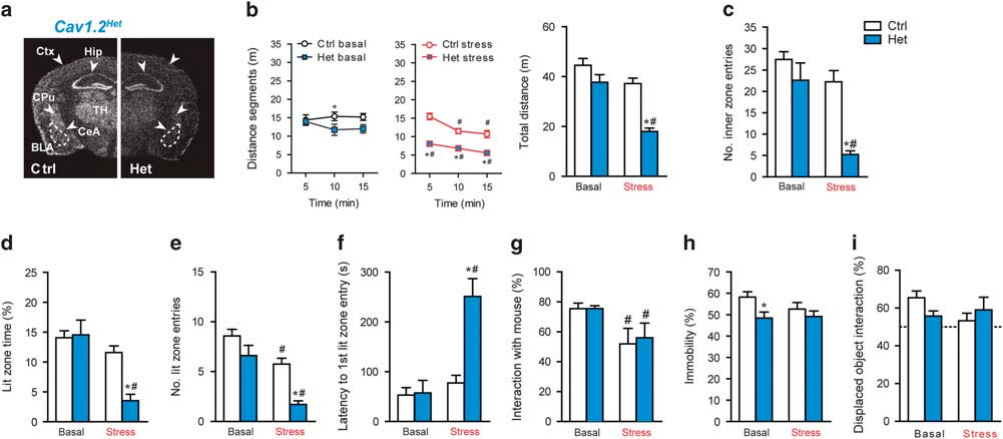
*Cacna1c* heterozygosis induces susceptibility to chronic social defeat stress (CSDS). (**a**) *Cacna1c in situ* hybridization (ISH) in control (Ctrl) and *Cav1.2*^*Het*^ (Het) mice, in which heterozygosis is present from the first day of development. (**b**) Locomotion (distance segments: time × genotype × stress, F_(2,41)_=4.3, *P*=0.02/total distance: genotype, F_1,42_=23.6, *P*<0.0001; stress, F_1,42_=25.4, *P*<0.0001; genotype × stress, F_1,42_=4.9, *P*=0.033) and (**c**) number of inner zone entries (stress × genotype, F_1,42_=5.1, *P*=0.03; stress, F_1,42_=18.1, *P*<0.0001; genotype, F_1,42_=16.3, *P*=0.0002) in the open field (OF) test (*n*=12 Ctrl basal, 15 Ctrl stress, 10 Het basal, 9 Het stress). (**d–f**) Dark/light box test (Lit zone time: stress × genotype, F_1,44_=8.40, *P*=0.006; stress, F_1,44_=21.2, *P*<0.0001; genotype, F_1,44_=6.6, *P*=0.01/Lit zone entries: stress, F_1,44_=26.1, *P*<0.0001; genotype, F_1,44_=16.3, *P*<0.0001; *n*=14 Ctrl basal, 15 Ctrl stress, 10 Het basal, 9 Het stress). (**g–i**) Sociability test (stress, F_1,35_=8.0, *P*=0.008), forced swim test (FST; genotype, F_1,35_=5.6, *P*=0.01) and spatial object recognition test (*n*=9 Ctrl basal, 10 Ctrl stress, 10 Het basal, 10 Het stress). Two-way analysis of variance (ANOVA)+Bonferroni *post hoc* test and repeated measures (RM)-ANOVA+Bonferroni *post hoc* test; *significantly different from the Ctrl group of the same condition, #significantly different from the basal condition of the same genotype. Data are means±s.e.m.

Chronic stress and/or trauma represent strong risk factors for a number of psychiatric disorders, including MDD, BPD and anxiety disorders such as post-traumatic stress disorder.^24, 25, 26, 57^ In view of the phenotype in *Cav1.2-Dev*^*Glu-CKO*^ mice, we wondered whether developmental deletion of *Cacna1c* would increase the susceptibility to CSDS. We choose to investigate heterozygous *Cacna1c* animals (*Cav1.2*^*Het*^, Figure 3a), considering that *Cav1.2-Dev*^*Glu-CKO*^ mice already display strong behavioral deficits under baseline conditions, which might not be further aggravated by CSDS. Importantly, no gross behavioral abnormalities were previously reported for *Cav1.2*^*Het*^ mice.^33^ *Cav1.2*^*Ctrl*^ and *Cav1.2*^*Het*^mice were subjected to 3 weeks of CSDS. In accordance with other studies,^41, 42^ we were able to detect robust physiological and neuroendocrine changes evoked by CSDS, independent of genotype, demonstrating the efficacy of the paradigm (Supplementary Figures S8a–d). Chronically stressed *Cav1.2*^*Het*^ mice displayed drastically reduced locomotion throughout the entire test duration compared to both, non-stressed *Cav1.2*^*Het*^ and stressed *Cav1.2*^*Ctrl*^ mice (Figure 3b). In addition, only chronically stressed *Cav1.2*^*Het*^ mice showed a significant reduction in the number of inner zone entries of the OF, indicating increased anxiety-related behavior even under low-light test conditions (Figure 3c). Moreover, CSDS increased the latency to enter the lit compartment and decreased the time spent in the lit zone and number of lit entries to a much greater extent in *Cav1.2*^*Het*^ than in *Cav1.2*^*Ctrl*^ mice (Figures 3d–f), further supporting increased stress-induced anxiety in heterozygous *Cacna1c* mice. Sociability, immobility in the FST and spatial object recognition memory were not differentially affected by CSDS (Figures 3g–i), suggesting a specific impact of the interaction between *Cacna1c* and chronic stress on anxiety.

**Figure 4:**
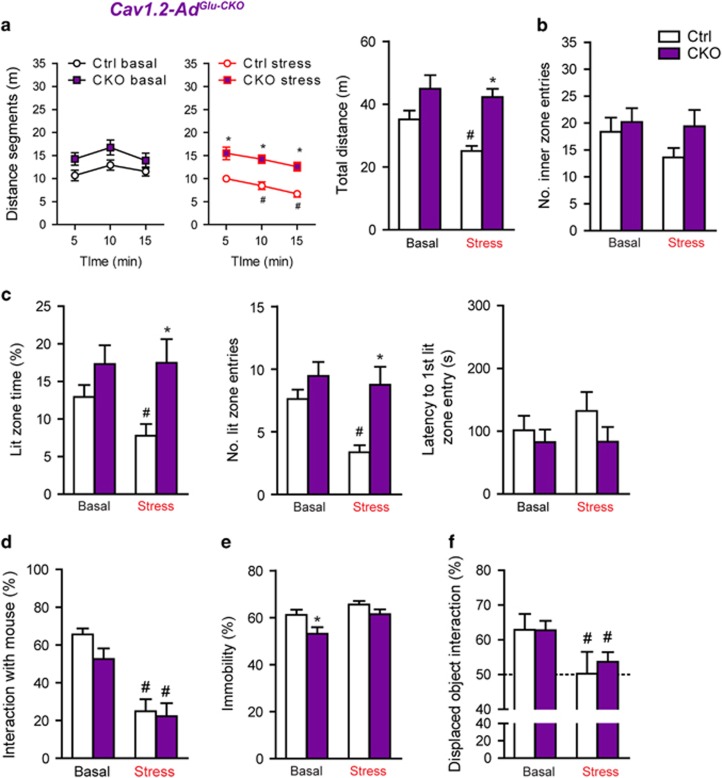
Deletion of *Cacna1c* from forebrain glutamatergic neurons during adulthood promotes resilience to chronic social defeat stress (CSDS). (**a**) Locomotion (distance segments: time × stress, F_2,45_=10.7, *P*<0.0001/total distance: genotype, F_1,45_=19.6, *P*<0.0001; stress, F_1,45_=4.3, *P*=0.04) and (**b**) number of inner zone entries in the open field (OF). (**c**) Dark/light box test (Lit zone time: genotype, F_1,45_=9.3, *P*=0.004/Lit zone entries: stress, F_1,45_=5.8, *P*=0.02; genotype, F_1,45_=12.3, *P*<0.001). (**d**) Sociability test (stress, F_1,45_=52.52, *P*<0.0001) and (**e**) forced swim test (FST; genotype, F_1,45_=7.5, *P*=0.009; stress, F_1,45_=8.3, *P*=0.006). (**f**) Spatial object recognition test (stress, F_1,42_=7.1, *P*=0.01). Two-way analysis of variance (ANOVA)+Bonferroni *post hoc* test and repeated measures (RM)-ANOVA+Bonferroni *post hoc* test; *significantly different from the control (Ctrl) group of the same condition, #significantly different from the basal condition of the same genotype. OF, dark/light box test, sociability and FST (*n*=13 Ctrl basal, 12 Ctrl stress, 12 conditional knockout (CKO) basal, 12 Het stress). Spatial object recognition test (*n*=12 Ctrl basal, 10 Ctrl stress, 12 CKO basal, 12 CKO stress). Data are means±s.e.m.

In view of the partially opposing effects of *Cacna1c* on anxiety and cognition during development and adulthood, we wondered whether stress vulnerability would also be differentially affected upon deletion of the calcium channel in adulthood. Physiological and neuroendocrine parameters were similarly altered by CSDS in Ctrl and *Cav1.2-Ad*^*Glu-CKO*^ mice (Supplementary Figures 8e–h). In contrast to *Cav1.2*^*Het*^ mice, locomotion/exploration in the OF was only decreased in stressed Ctrl but not stressed *Cav1.2-Ad*^*Glu-CKO*^ mice, with a similar trend in the number of inner zone entries (Figures 4a and b). In the dark/light box test, only Ctrl mice responded to CSDS with a significant reduction in time spent in the lit zone and number of entries, and a trend toward delayed latencies (Figure 4c), which implies enhanced resilience to CSDS in *Cav1.2-Ad*^*Glu-CKO*^ mice. FST behavior, sociability and spatial object were similarly affected by CSDS in Ctrl and *Cav1.2-Ad*^*Glu-CKO*^ mice (Figures 4d–f). Considering that we observed susceptibility to CSDS in heterozygous Ca_v_1.2 mice, we additionally assessed whether a heterozygous deletion in glutamatergic neurons during adulthood (*Cav1.2-Ad*^*Glu-Het*^) would be sufficient to induce stress resilience (Supplementary Figure S9). However, compared with *Cav1.2-Ad*^*Glu-CKO*^, *Cav1.2-Ad*^*Glu-Het*^ mice only exhibited partial resilience to CSDS-induced anxiety in the dark/light box test (Supplementary Figure S9f). This points to a gene-dosage effect and suggests that complete absence of *Cacna1c* during adulthood, in forebrain glutamatergic neurons, is required to induce the extent of resilience to CSDS observed in *Cav1.2-Ad*^*Glu-CKO*^mice.

### *CACNA1C* interacts with adult trauma to predict depression symptoms in humans

**Figure 5:**
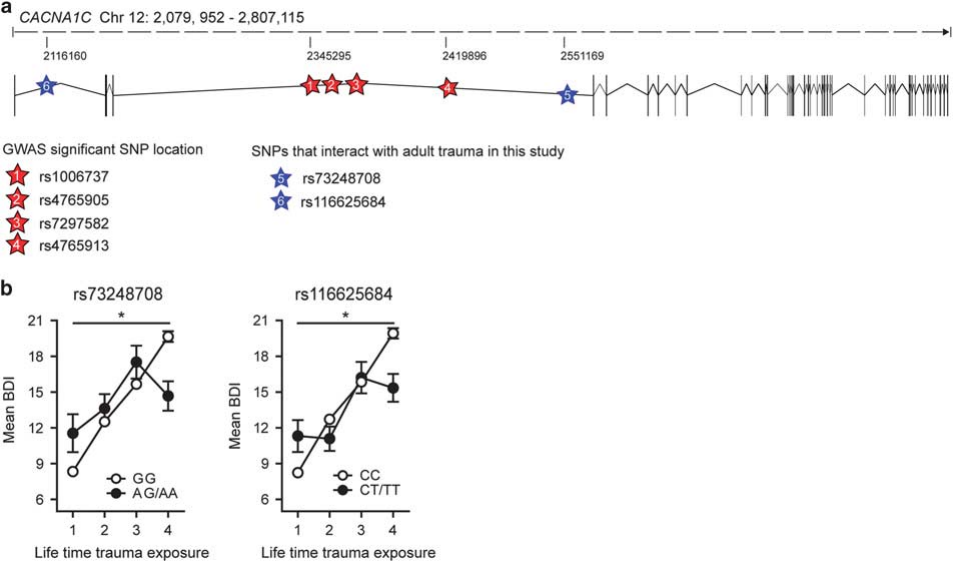
*CACNA1C* interacts with adult trauma to predict depressive symptoms in humans. (**a**) Single-nucleotide polymorphism (SNP) localization sites in the human *CACNA1C* locus. Exons are indicated with vertical black bars. (**b**) rs73249708 (*P*=0.0004) and rs116625684 (*P*=0.0016) significantly interacted with adult trauma to predict Beck Depression Inventory scores. Asterisk indicate an interaction *P*-value below 0.0016, hence surviving Bonferroni correction. The phenotype on adult trauma was categorized into its four quartiles to facilitate interpretation of the plot. Sample size for individual genotypes: rs73248708 (*n*=4106 GG, 331 AG, 7 AA), rs116625684 (*n*=4372 AA, 367 AG, 7 GG). Data are means±s.e.m.

A number of *CACNA1C* SNPs associated with BPD, SCZ and MDD were also shown to affect measures of anxiety, depression, psychosocial functioning and cognitive aspects in healthy Ctrls,^58^ which might represent increased susceptibility factors for psychiatric disorders upon exposure to adverse environments. This is especially interesting in the context of MDD, in which disease susceptibility is strongly influenced by previous exposures to chronic and/or severe stress. Considering that most GWAS do not Ctrl for life-time adversity, we wondered whether certain *CACNA1C* SNPs might primarily influence the risk for MDD in combination with stressful life events. Consequently, we analyzed the effects of all *CACNA1C* variants and adult trauma on current depression symptoms, determined by the Beck Depression Inventory^59^ in a large African American cohort of non-psychiatric clinical patients (*n*=4808) from the Grady trauma project.^49, 50, 60^ Importantly, symptoms of depression are not only characteristic for MDD and BPD but can also occur as part of affective dysregulation in SCZ24. After linkage disequilibrium-pruning based on *r*^2^-values of 0.2, 32 of the 465 tested SNPs in the CACNA1C locus showed nominal significant interactions with adult trauma exposure on Beck Depression Inventory scores. Of these, two remained significant after Bonferroni correction for multiple testing; rs73248708, nominal *P*=1.38 × 10^−5^, Bonferroni corrected *P*=0.0004; rs116625684, nominal *P*=5.2 × 10^−5^, Bonferroni corrected *P*=0.0016 (Figures 5a and b, and Supplementary Table S1 and S2). Both SNPs also showed nominal significant main effects (rs73248708: nominal *P*=1.88 × 10^−4^, rs116625684: nominal *P*=0.02). Interestingly, in both cases, individuals carrying at least one minor allele (CT/TT or AG/AA) and without trauma history displayed significantly higher Beck Depression Inventory scores compared with the major allele group, whereas the opposite was observed for minor allele carriers with the highest degree of lifetime trauma exposure.

## Discussion

Our results demonstrate that spatial memory, hippocampal plasticity and anxiety-related behavior are differentially affected by the loss of *Cacna1c* from excitatory circuits depending on whether it is deleted during embryonic development or adulthood. Increased anxiety-related behavior as well as reduced social and cognitive performance, observed in *Cav1.2-Dev*^*Glu-CKO*^ mice, are considered core endophenotypes of MDD and/or the depressive phase of BPD,^52, 53^ whereas increased locomotion and activity in the FST are often associated with mania in animal models of BPD.^53, 55^ Decreased immobility in the FST was previously reported for heterozygous mice by Dao *et al.*,^33^ which is in line with our results and earlier work demonstrating antidepressant-like effects for LTCC blockers.^27, 61^ However, studies using LTCC blockers have to be interpreted with caution, as these have been reported to induce nonspecific, aversive and stress-like behavioral effects possibly due to excessive blood pressure lowering caused by inhibition of Cav1.2 in the cardiovascular system.^62, 63, 64^ Importantly, hyperlocomotion, altered social behavior, and learning and memory impairments, are also analogous to negative and cognitive symptoms of SCZ, a disorder believed to have neurodevelopmental origins.^24, 54^ Additional evidence also suggests that two missense mutations in CACNA1C predispose to autism,^65, 66^ another neurodevelopmental disorder characterized by impaired social interactions and altered cognition. In accordance with the observed cognitive impairments, *Cav1.2-Dev*^*Glu-CKO*^ mice also displayed deficits in hippocampal LTP, which is considered the cellular correlate of learning and memory.^67^ Moreover, we show that *Cacna1c* strongly interacts with the environment to shape anxiety-related behavior. Heterozygous deletion of *Cacna1c*, which is present throughout life starting at the earliest point of development (one-cell stage), significantly increased the susceptibility to CSDS and is likely to be mediated by *Cacna1c* absence in forebrain glutamatergic neurons. Although *CACNA1C* SNPs have not been consistently associated with anxiety in humans, altered anxiety-related behavior is considered a core endophenotype of MDD and BPD in animal models.^52, 53, 55^ Different to our observations, a recent study showed that chronic unpredictable stress induces similar behavioral deficits in heterozygous Ca_v_1.2 mice and their wild-type littermates when assessed 1–2 days post stress. Surprisingly, the stress effects persisted in wild-type mice when tested 5–7 days later but not in heterozygous knockout mice.^68^ These discrepancies might be related to the different chronic stress paradigms.

In contrast to a developmental inactivation, deletion of *Cacna1c* from forebrain glutamatergic neurons during adulthood blocked the adverse effects of CSDS on anxiety, enhanced LTP and improved hippocampus-dependent cognitive flexibility during the WCM, without affecting long-term memory. Our findings in *Cav1.2-Dev*^*Glu-CKO*^ mice are supported by previous studies, showing that deletion of *Cacna1c* from pan-neuronal^37^ or glutamatergic neurons during development^29^ impairs synaptic plasticity and/or spatial learning, some of which might be attributed to deficits in adult neurogenesis.^37^ However, it should be kept in mind that our LTP results cannot be entirely correlated with cognitive performance considering that we stimulated CA3-CA1 Schaffer collaterals, which leads to the potentiation of most CA3-CA1 synapses rather than those that would selectively be recruited during a learning and memory task. Electrophysiological and pharmacological data have indicated that LTP induction is mainly initiated through NMDAR-activity but can also include NMDAR-independent and VGCC-dependent mechanisms.^69, 70^ Interestingly, the decline of some memory processes is attributed to the shift from NMDAR-dependent to VGCC-intracellular calcium stores-dependent regulation of synaptic plasticity, especially during aging.^71, 72, 73^ Along these lines, a recent study provides evidence for an association between increased hippocampal *Cacna1c* expression and age-related cognitive decline.^74^ Although we did not assess aged animals, it can be speculated that ablation of Ca_v_1.2 in adulthood partially blocks an evolving shift towards VGCC- and/or intracellular calcium store-dependent regulation of synaptic plasticity, hence facilitating NMDAR-dependent LTP. This potentially suggests that Hip-dependent spatial memory formation requires Ca_v_1.2 during prenatal development, but not in adulthood where activation of Ca_v_1.2 channels is even detrimental for LTP. Notably, calcium channel antagonists were shown to ameliorate age-related deficits in hippocampus-dependent memory, possibly mediated through facilitation of NMDAR-LTP or inhibition of long-term depression.^71, 73, 75, 76, 77, 78^ On the other hand, specific inactivation of *Cacna1c* in the anterior cingulate between postnatal weeks 8–10, impaired observational fear learning without altering novel object recognition memory and classical fear conditioning,^30^ highlighting the requirement for Ca_v_1.2 during adulthood for specific cognitive functions. The fact that we observed no significant changes in spatial object memory performance under basal or stress conditions further implies that adult-specific *Cacna1c* deletion in excitatory circuits does not impact all cognitive domains.

As observed in *Cav1.2-Dev*^*Glu-CKO*^ mice, locomotion was also enhanced in *Cav1.2-Ad*^*Glu-CKO*^ animals; however, social and active stress-coping behavior were only mildly affected, suggesting that these endophenotypes are partly regulated by forebrain-Ca_v_1.2 during development and adulthood. In contrast, an earlier study demonstrated that forebrain-specific elimination of *Cacna1c*, using a non-inducible *Camk2α-Cre*, also results in increased anxiety-like behavior.^34^ The discrepancy could potentially be attributed to different *Cacna1c* deletion time windows (p18^79^ vs p91-97 in our study).

Although the Nex-Cre and Camk2α-CreER^T2^ drivers primarily target glutamatergic forebrain neurons, they do slightly differ in their recombination patters (for example,the Camk2α-driven CreER^T2^ is also expressed in parts of the thalamus, DG and central nucleus of the amygdala). However, the fact that developmental, CNS-specific *Cacna1c* knockout mice showed similar behavioral deficits as *Cav1.2-Dev*^*Glu-CKO*^ mice, further supports opposing roles for Ca_v_1.2 during development and adulthood. Nevertheless, we cannot rule out an important contribution of Ca_v_1.2-expressing, GABAergic neurons in many of the disease-associated phenotypes. In fact, *Cacna1c* was reported to modulate processes related to drug addiction via GABAergic medium spiny neurons in the nucleus accumbens,^80, 81^ and promote susceptibility to social stress when deleted from this specific brain region.^82^ It is important to point out that we only assessed a subset of behavioral tests relevant to MDD, BPD and SCZ in our knockout mice. Future studies evaluating anhedonia, psychostimulant-induced locomotion, sleep architecture and deficits in sensorimotor gating (prepulse inhibition deficits), will further refine the contribution of developmental vs adult Ca_v_1.2 in psychiatric disorders.

Given our observations in mice, we further investigated if *Cacna1c* x stress interactions could also be found in human samples and checked for interaction effects of *CACNA1C* variants and adult trauma to predict current depressive symptoms

As expected, the risk to develop depressive symptoms was increased with exposure to adult trauma in our study and this effect was moderated by loci within CACNA1C tagged by rs73248708 and rs116625684. Both tag SNPs were not in linkage disequilibrium with the main haplotypes associated with SCZ with genome-wide significance, but represent independent loci. The intron 1 SNP rs116625684 shows a weak, nominal association with SCZ in the Psychiatric Genomics Consortium data set (*P*=0.021), with an odds ratio of 0.94 (http://www.med.unc.edu/pgc), suggesting a protective effect of the minor allele, which is also indicated by our results for the highest trauma group. The second SNP, rs73248708 is located in intron 3, which harbors the majority of *CACNA1C* SNPs associated with SCZ at genome-wide significance and also those linked to other disorders including BPD and MDD.^19^ In fact, SNPs in this region were shown to lie in predicted enhancers capable of physically interacting with the *CACNA1C* transcriptional start site, and altering gene expression.^83^ Although rs73248708 is not in linkage disequilibrium with the top GWAS SNPs, including rs1006737, it may independently moderate the function of this enhancer region. In fact, we could show that different sets of genetic variants may be involved in baseline transcriptional regulation vs. regulation following environmental impact, such as trauma exposure.^84^ Replication in independent cohorts will be necessary to further validate these loci in gene × environment interactions. Importantly, it remains to be investigated whether the here identified SNPs have implications on protein levels or are of other functional relevance. So far, both decreased and increased Ca_v_1.2 levels have been associated with the common risk allele variant rs1006737,^85, 86, 87^ which might imply different regulatory functions across different brain regions and/or cell types. Whether the here identified SNPs, as well as previously associated *CACAN1C* risk alleles, can differentially affect gene expression in the brain upon perturbations such as severe stress and/or trauma will have to be assessed in the future. Considering the heterogeneity of MDD, our data raises the possibility that SNPs in CACNA1C might predict the risk for depression mainly in individuals with a history of trauma or severe stress. This could partially explain the nominal associations of CACAN1C with MDD in former genetic studies, which usually do not Ctrl for environmental factors. In addition, given our animal findings it would be interesting to examine for age × trauma interaction effects in the human sample. However, in the present cohort we did not have any information with regard to when exactly adult trauma took place and therefore cannot evaluate age effects.

There are some limitations of the interpretability of our findings, which should not be neglected. First, the Grady Trauma Project includes mostly African Americans. We cannot rule out that effects of *CACNA1C* variants are different in this specific population as compared with the European population where the effect of *CACNA1C* variants with regard to MDD were described first. Second, the GRADY sample itself is a highly traumatized cohort. It is possible that the described effects are only present in samples which show a high trauma burden and cannot be found in less traumatized cohorts. However, findings in this cohort can help to transit the results from mice to human data.

Although circuit- or cell type-specific absence of CACNA1C is not likely to occur in humans, epigenetic changes caused by environmental alterations may very well induce expression changes in specific tissues, cell types and/or entire brain circuits. In the clinical context this could imply that, individuals with genetic variants promoting reduced CACNA1C expression may either be at higher or lower risk to develop psychopathologies, depending on whether they experienced severe stress/trauma during development or adulthood. The pronounced effect of a prenatal *Cacna1c* depletion on emotional behavior and cognitive performance might account for its association with disorders such as SCZ and autism, which are increasingly believed to result from synaptic dysfunction during development. The fact that *CACNA1C* is also able to interact with the environment during adulthood, by modulating stress susceptibility, could explain its linkage to stress-related disorder such as MDD and BPD. At the same time, Ca_v_1.2 and Ca_v_1.3 are expressed in midbrain dopaminergic neurons and several studies suggest a neuroprotective effects of LTCC blockers in Parkinson’s disease,^22, 88^ which further emphasizes the possibility that inhibition of Ca_v_1.2 may be of therapeutic value in brain disorders that manifest at later stages in life. Concluding, we propose that the association of *CACNA1C* with multiple psychiatric disorders is related to its broad expression within key limbic regions and neuronal circuits relevant to emotion, motivation and cognition, and that alterations in *CACNA1C* gene expression during development and adulthood can result in diverging behavioral outcomes and differentially impact stress susceptibility.
