# Supplementary material for: Cross-disorder risk gene CACNA1C differentially modulates susceptibility to psychiatric disorders during development and adulthood
Source: Mol Psychiatry. 2017 Jul 11;23(3):533–43. doi: 10.1038/mp.2017.133 (PMC5822460; doi:10.1038/mp.2017.133)
Supplement: Supplementary Information [file mp2017133x1.docx]

**Supplementary Information**

Supplementary Material and Methods

Supplementary Figures S1-S7

Supplementary Tables S1-3

**Supplementary Material and Methods**

**Western blot analysis**

Tissue was lysed in RIPA buffer containing protease inhibitors (Roche Diagnostics, Indianapolis, IN), 20 µM NEM 1,10-OPT (Sigma-Aldrich, St. Louis, MO). Protein samples were separated by 8% SDS-PAGE and transferred to 0.45-µm PVDF membranes (EMD Millipore, Billerica, MA). The membranes were then incubated with the Ca_v_1.2 primary antibody^29^ and a secondary HRP-conjugated antibody. Chemiluminescence signals were visualized in a ChemiDoc station (Bio-Rad Laboratories Inc., Hercules, CA) and analyzed using Image Lab (Bio-Rad Laboratories Inc., Hercules, CA). Three independent experiments were performed for all Western blots.

**Chronic social defeat stress (CSDS) paradigm**

Experimental mice (9-13 male mice per group between 3-4 months of age) were submitted to CSDS for 21 consecutive days. They were introduced into the home cage (45 cm x 25 cm) of a dominant CD1 resident for no longer than 5 min, and were subsequently defeated. Following defeat, animals spent 24 hours in the same cage, which was separated via a perforated steel partition, enabling sensory but not physical contact. Every day experimental mice were exposed to a new unfamiliar resident. Defeat encounters were randomized, with variations in starting time in order to decrease the predictability to the stressor and minimize habituation effects. Control animals were housed in their home cages throughout the course of the experiment. Control and stressed mice were housed in the same room, but in different racks. All animals were handled daily; weight and fur status were assessed every 3-4 days. Behavioral testing was conducted during the last week of the CSDS paradigm.

**Open field (OF) test**

The OF test was used to characterize locomotor activity in a novel environment. Testing was performed in an open field arena (50 x 50 x 50 cm) dimly illuminated (about 15 lux) in order to minimize anxiety effects on locomotion. All mice were placed into a corner of the apparatus at the beginning of the trial. The distance traveled and time spent in the outer and inner zones was assessed with the ANY-maze software (4.20; Stoelting).

**Dark/light box test**

The dark/light box test was performed in a rectangular apparatus (15 x 20 x 25 cm) consisting of an aversive brightly lit compartment (700 lux) and a more protective dark compartment (5 lux). At the start of the test, all mice were placed in the dark compartment and were allowed to freely explore the apparatus for 5 min. Lit zone entries were counted if at least the two front paws and half of the animal’s body were inside the lit compartment. Automatic tracking was employed using the ANY-maze (4.20; Stoelting).

**Sociability test**

The sociability test was performed using a three chamber apparatus, as previously described^41,44^. Briefly, during the sociability trial an unfamiliar male C57BL/6J mouse was introduced into one of the chambers, enclosed in a wire cage, while a toy mouse was placed in the opposite chamber (alteration occurred after 3 consecutive trials). The time spent interacting with mouse and object was scored for 10 minutes by a trained observer.

**Spatial object recognition memory task**

Spatial object memory was assessed in the OF arena under low illumination (10 lux). During the acquisition trial, mice were presented with two identical objects (salt shakers) and allowed to freely explore the objects for 10 min. Following a 30 min inter-trial interval, mice were presented with a nondisplaced object and a relocated one. Spatial cues were provided during both trials. The percentage of time exploring the displaced objects was calculated. A higher preference for the displaced object reflects intact spatial recognition memory. Mice that did not interact with either of the object were excluded from the test.

**Water-cross maze (WCM)**

Similarly to the classical Morris water maze, the WCM makes use of water-based motivation, but additionally allows to clearly discriminate between different learning strategies^46^. In addition, it employs a direct measure of accuracy rather than just escape latency, which is often affected by non-cognitive factors such as alterations in locomotion or motivation. The maze consists of two intersecting arms, forming a cross, made from clear acrylic to enable visual orientation within the room. A submerged platform was located in one of the arms, 1 cm under the water surface, invisible to the mice. Every animal performed six trials a day for five consecutive days. During this time the platform was always located in the same arm (for example East), whereas the starting position of the mice alternated between South and North in a pseudorandom manner. The latency to reach the platform was set to 1 min. Learning performance was assessed by the accuracy.

Accuracy: A trial was considered accurate (i.e., value 1), if the animal directly entered the arm containing the platform and climbed onto it. Aberrant behavior was considered as non-accurate (i.e., value 0). Thus, accuracy reflects the percentage of accurate trials on each day per animal. An animal reached the criterion of an accurate learner, if it accomplished more than 83% accurate trials per day (i.e., ≥5 out of 6 trials).

**Long-term potentiation (LTP) recordings**

Mice were anesthetized with isoflurane and decapitated shortly after. The brains were removed and quickly transferred into ice-cold carbogenated (95% O_2_/5% CO_2_) artificial cerebrospinal fluid (aCSF). Sagittal hippocampal slices (350µm) were obtained using a vibratome (HM 650V, Microm International, Walldorf, Germany). The slices were allowed to recover for at least 1h at room temperature before being transferred to the recording chamber where they were continuously superfused with aCSF at a rate of 5ml/min. The aCSF contained (in mM): NaCl, 125; KCl, 2.5; NaHCO_3_, 25; CaCl_2_, 2; MgCl_2_, 1; D-glucose, 25; NaH_2_PO_4_, 1.25, and was saturated with a mixture of 95% O_2_/5% CO_2_, final pH 7.3. Field excitatory postsynaptic potentials (fEPSPs) at synapses between Schaffer collateral-commissural pathway (SCCP) and CA1 pyramidal cells were recorded in the stratum radiatum of the CA1. High-frequency stimulation (HFS 100 Hz/100 pulses) to the SCCP was delivered to induce LTP. The recordings were amplified, filtered (1 kHz) and digitized (3 kHz) using a laboratory interface board (ITC-16, InstruTech, Longmont, CO), and stored with the acquisition program Pulse, version 8.5 (Heka Elektronik, Lambrecht, Germany). Data were analyzed offline with the analysis program IgorPro v.6.1 software (WaveMetrics, Inc., Portland, OR). Measurements of the initial slope of the fEPSP were taken and normalized with respect to the 10 min baseline period before HFS.

**Trauma events inventory (TEI)**

The TEI in this study assesses lifetime history of trauma exposure to a range of traumatic events excluding childhood abuse^51,88^ and used as semi-quantitative variable in the GXE analysis. For graphical representation of the results individuals were grouped according to quartiles of trauma exposure severity. Although the TEI also includes exposure to non-child abuse traumatic events in childhood, the mean age of exposure was 20, thus mostly referring to adult trauma.

**Beck depression inventory (BDI)**

The 21-item self-report BDI^59^ was applied in this study to measure severity of current depressive symptoms.

**Gene** × **environment analysis in humans**

The genotype x environment interaction analysis was performed on genotypes derived from the Omni express v2 and Omni 1M arrays. All experimental procedures were performed according to the manufacturer’s protocol. SNPs with a call-rate below 98%, a minor allele frequency below 1% or deviating from Hardy-Weinberg-Equilibrium (p<0.000001) were excluded from further analysis. Four individuals were removed as their heterozygosity rate deviated more than 5 SD from the mean heterozygosity. We imputed this dataset (635,983 SNPs and 4,808 individuals) with the impute2 (https://mathgen.stats.ox.ac.uk/impute/impute_v2.html) and shapeit2 (https://mathgen.stats.ox.ac.uk/genetics_software/shapeit/shapeit.html) software and using the 1000 Genomes Phase I data as reference set. After imputation, we re-ran a quality control excluding SNPs deviating from Hardy-Weinberg-Equilibrium (p<0.000001) or presenting with an info metric < 0.8. Imputation probabilities were recoded as best-guessed-genotypes using PLINK2 and setting a hard-call threshold of 0.90 (https://www.cog-genomics.org/plink2). The minor allele frequencies of the 2 top SNPs were as follows: rs73248708 – 0.039 and rs116625684 - 0.04. Effects of *CACNA1C* SNPs and adult trauma (i.e. SNP x trauma interaction) on BDI were assessed using generalized regression models (additive genotype effect) in R (https://www.r-project.org/). We included age, gender and the first two population principal components for ancestry as covariates in the analysis. The results were corrected for multiple testing using the Bonferroni threshold (p = 0.05/465 = 0.0001) of significance.

**Supplementary Figures**

**
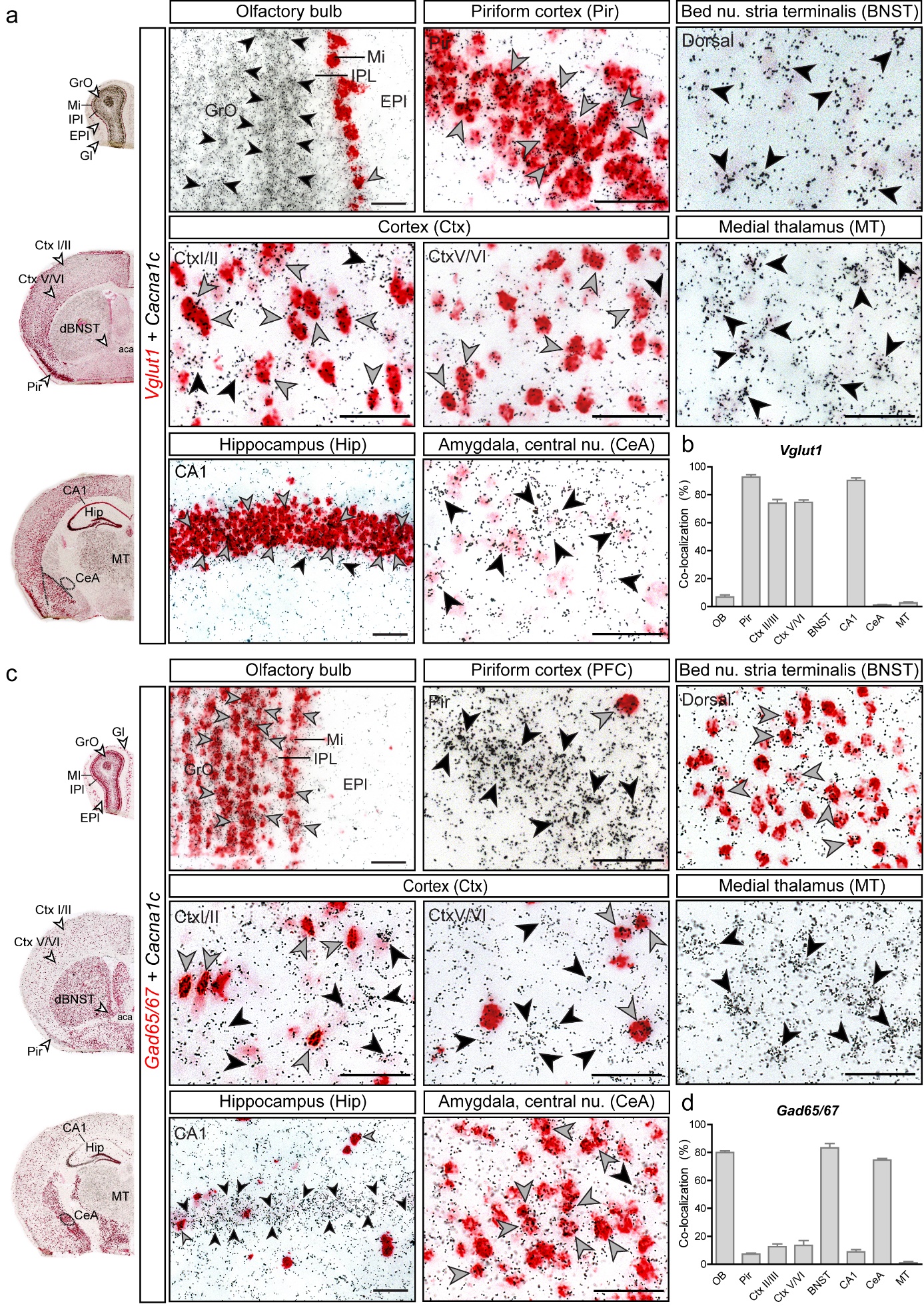
**

**Figure S1.** **Neurotransmitter identity of *Cacna1c*-expressing neurons**

**
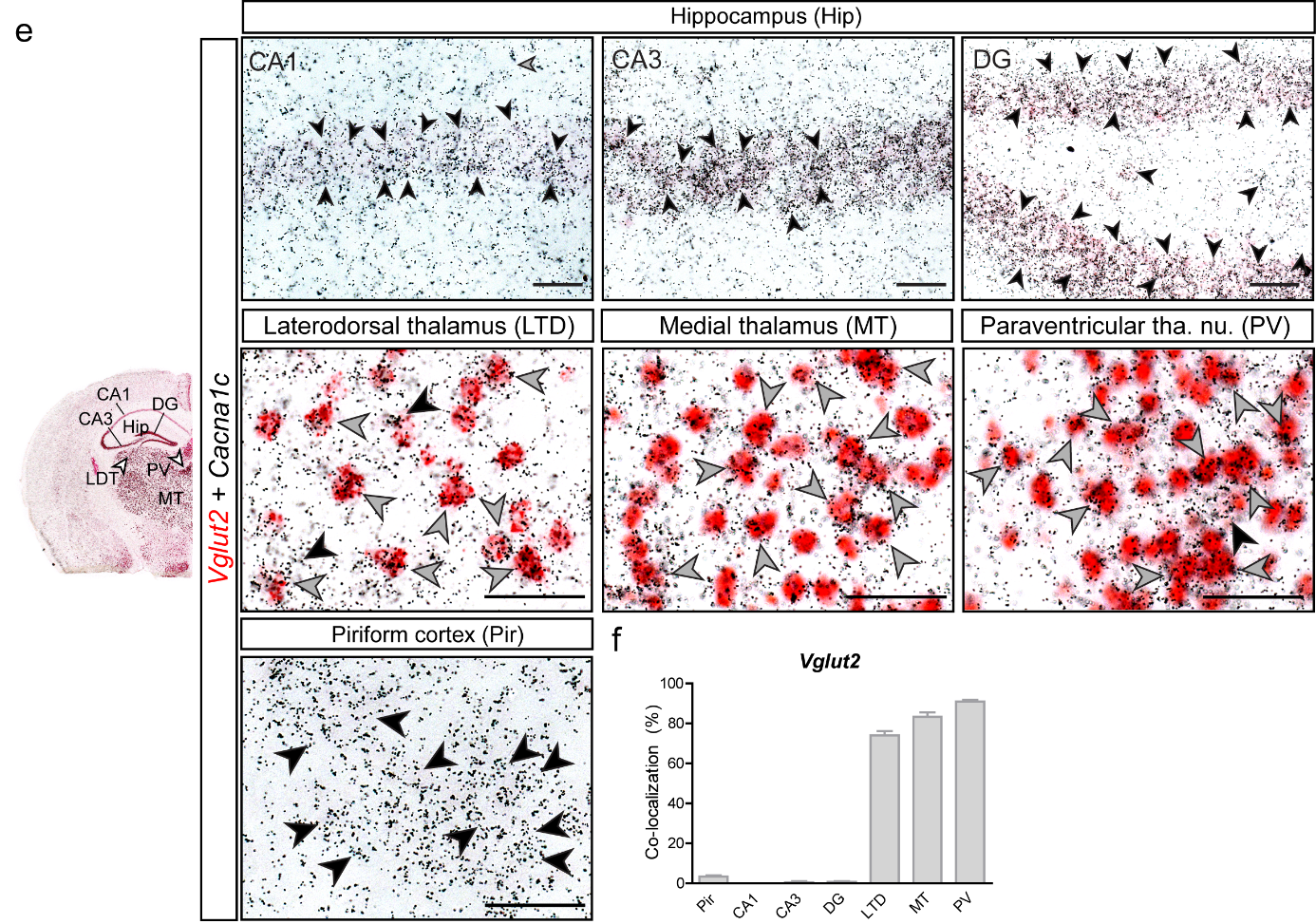
**

**Figure S1 continued**. **Neurotransmitter identity of *Cacna1c*-expressing neurons.**

(**a**) Double *in situ* hybridizations revealed a pronounced expression of *Cacna1c* (silver grains) in glutamatergic (*Vglut1*) neurons throughout all cortical layers including the piriform cortex, as well as the CA1 of the hippocampus. Minimal to no co-expression was detected in the olfactory bulb, bed nucleus of the stria terminalis (BNST), central nucleus of the amygdala (CeA) and medial thalamus (MT). (**b**) Quantifications of *Cacna1c* co-expression with *Vglut1* (n = 3, 2-3 sections/mouse). (**c**) Within the olfactory bulb, BNST and CeA, *Cacna1c* is predominantly expressed in GABAergic (Gad65/67) neurons. *Cacna1c* was also detected in a number of cortical GABAergic neurons. Minimal to no co-expression was observed for the CA1 of the hippocampus, piriform cortex and MT. (**d**) Quantifications of *Cacna1c* co-expression with *Gad65/67* (n = 3, 2-3 sections/mouse). (**e**) The prominent Cacna1c expression throughout the thalamus is largely restricted to glutamatergic *Vglut2*-positive neurons. The absence of *Vglut2*-positve *Cacna1c* neurons in the piriform cortex and hippocampus confirms the largely mutually exclusive expression patterns of *Vglut1* and *Vglut2*. (**f**) Quantifications of *Cacna1c* co-expression with *Vglut2* (n = 3, 2-3 sections/mouse). Black arrowheads indicate cells only expressing *Cacna1c*. Gray arrowheads indicate cells co-expressing *Cacna1c* and the respective neurotransmitter marker (red staining). Scale bars represent 50 µm. Abbreviations: Dentate gyrus (DG), External plexiform layer of the olfactory bulb (EPl), glomerular layer of the olfactory bulb (Gl), granule cell layer of the olfactory bulb (GrO), internal plexiform layer of the olfactory bulb (IPL), mitral layer of the olfactory bulb (Mi).

**
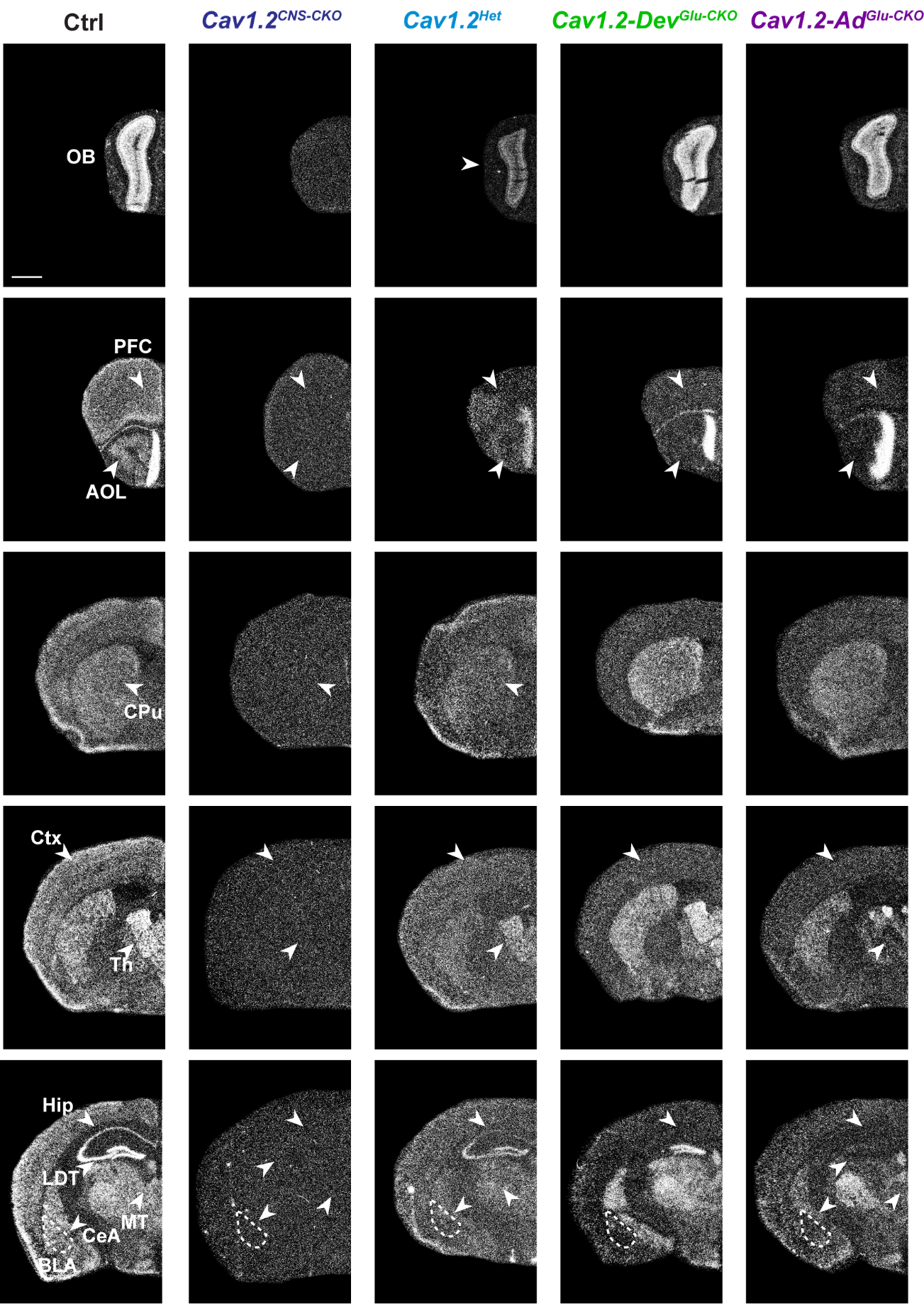
**

**Figure S2. Specific deletion pattern of *Cacna1c* mRNA throughout the brain of different conditional *Cav1.2^CKO^* mouse lines.**

**
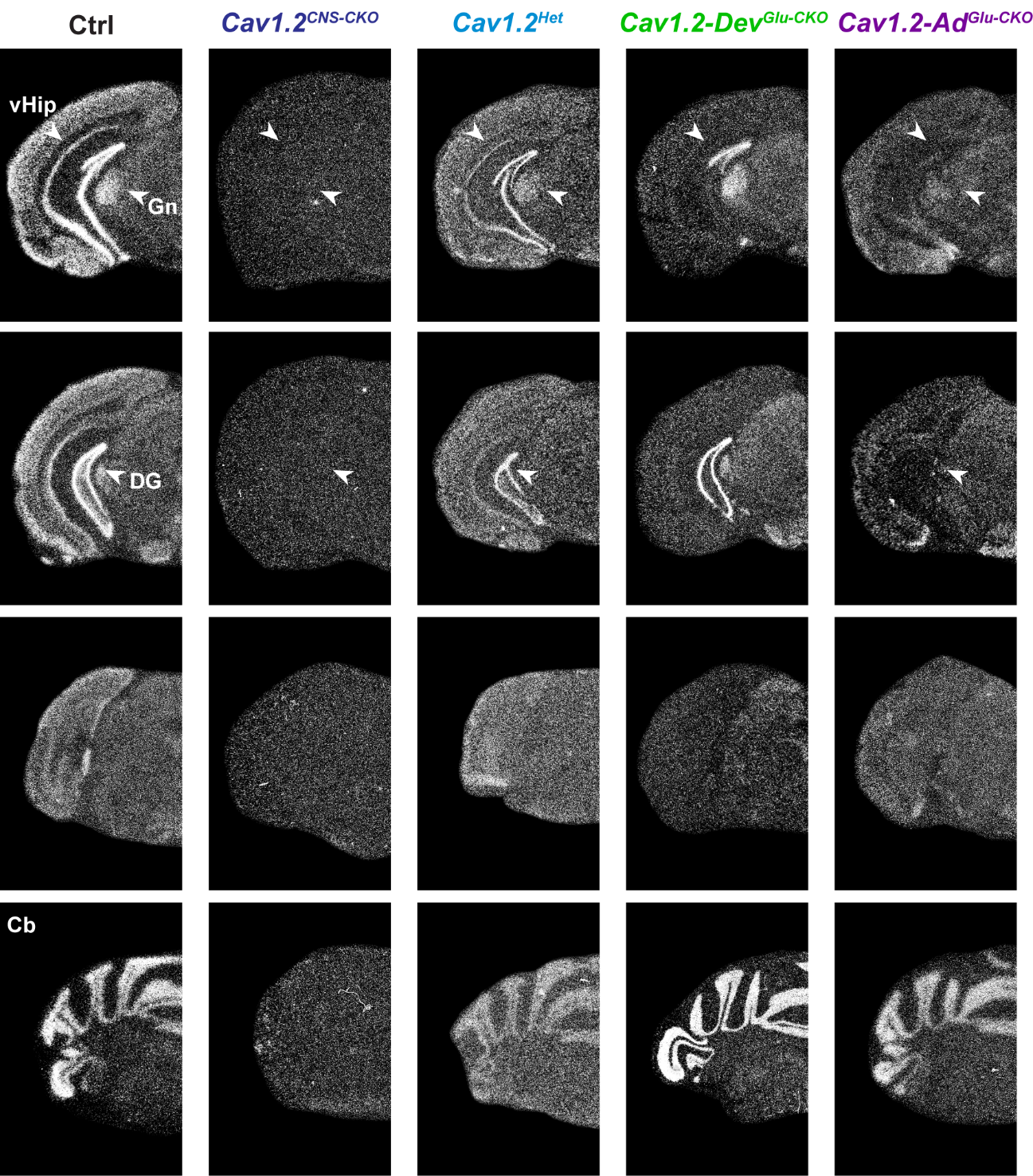
**

**Figure S2 continued. Specific deletion pattern of *Cacna1c* mRNA throughout the brain of different conditional *Cav1.2^CKO^* mouse lines.** Representative dark-field photomicrographs of radioactive *in situ* hybridizations show *Cacna1c* mRNA expression in coronal brain sections of control *Cav1.2^CNS-CKO^*, *Cav1.2^Het^*, *Cav1.2-Dev^Glu-CKO^* and *Cav1.2-Ad^Glu-CKO^* mouse lines. Noticeable areas of *Cacna1c* deletion compared to Ctrls are highlighted with arrowheads. Specific deletion of the *Cacna1c* gene in forebrain glutamatergic neurons (*Cav1.2-Dev^Glu-CKO^*) renders complete loss of *Cacna1c* mRNA expression in the cerebral cortex (Ctx), hippocampal formation (Hip), and lateral divisions of the amygdala including the basolateral nucleus of the amygdala (BLA). A similar deletion pattern was observed in *Cav1.2-Ad^Glu-Ctrl^* mice, lacking *Cacna1c* in forebrain *Camk2α*-positive principal neurons; additional signal loss was also observed in the dentate gyrus (DG), the latero-dorsal and medial thalamus (LDT and MT), and a few cells of the caudate putamen (CPu), central nucleus of the amygdala (CeA) and geniculate nucleus of the midbrain (Gn). Additional abbreviations: Anterior olfactory area (AOL), caudate putamen / striatum (CPu), cerebellum (Cb), prefrontal cortex (PFC), olfactory bulb (OPB), ventral hippocampus (vHip). Scale bar represents 1 mm.

**
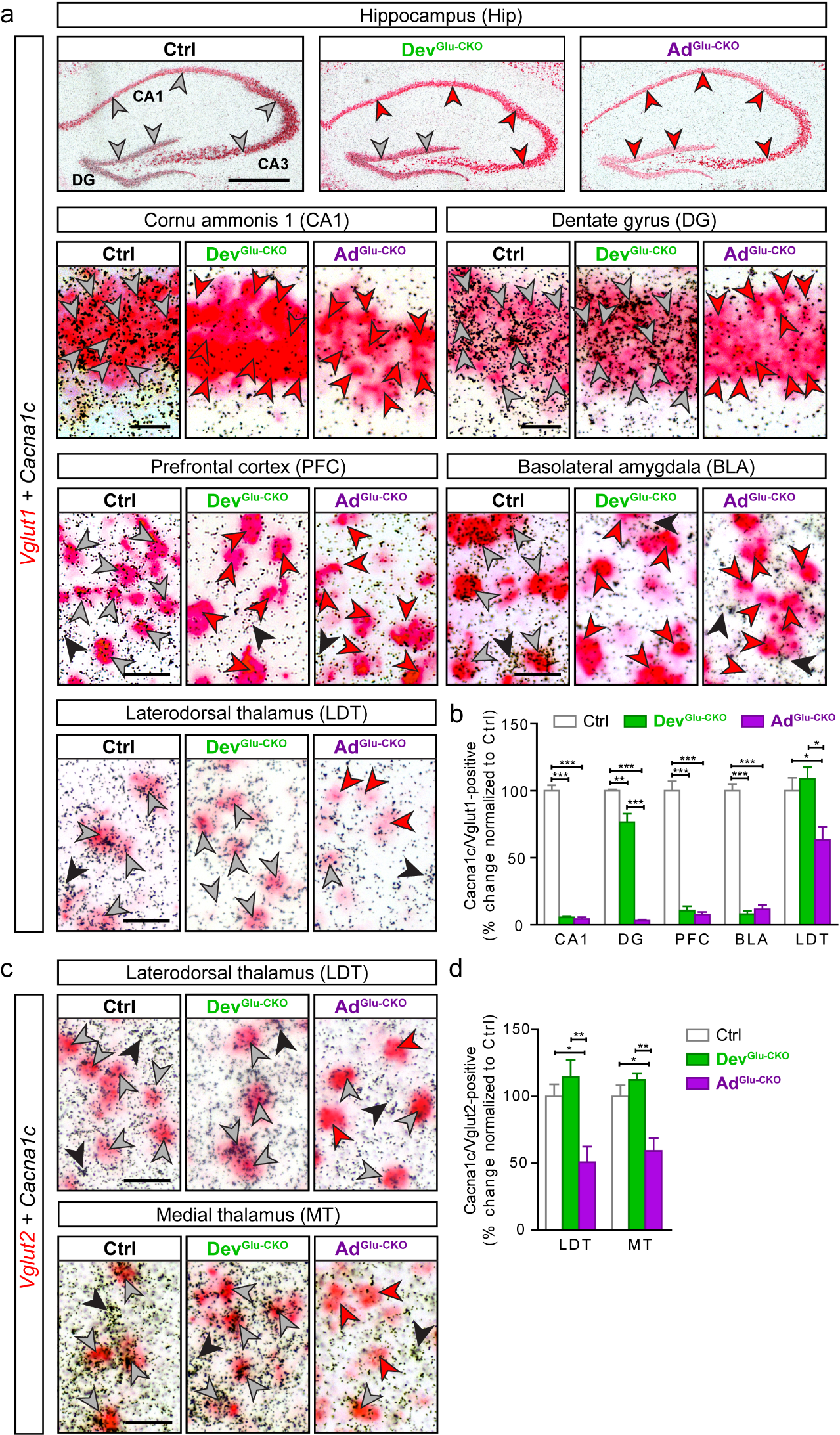
**

**Figure S3. *Cav1.2-Dev^Glu-CKO^* and *Cav1.2-Ad^Glu-CKO^* mice exhibit specific loss of *Cacna1c* in glutamatergic neurons.**

(**a**) Double ISHs in *Cav1.2-Dev^Glu‑CKO^* and *Cav1.2-Ad^Glu-CKO^* mice demonstrated absence of *Cacna1c* mRNA expression (silver grains) in *Vglut1*-positive, glutamatergic neurons (red staining) across different brain regions. Deletion of *Cacna1c* expression in *Vglut1* positive neurons was observed in the hippocampal regions CA1 and CA3 of *Cav1.2-Dev^Glu‑CKO^* and *Cav1.2‑Ad^Glu‑CKO^* mice. Absence of *Cacna1c* in *Vglut1* positive neurons was observed throughout the DG of *Cav1.2-Ad^Glu‑CKO^* mice whereas deletion in *Cav1.2-Dev^Glu‑CKO^* mice was only minimal. In the PFC and BLA, *Cacna1c* was deleted in glutamatergic neurons to a similar extent in both mouse lines. In contrast, deletion of *Cacna1c* from a subset of *Vglut1* neurons of the LDT was only detected in *Cav1.2‑Ad^Glu-CKO^* but not in *Cav1.2-Dev^Glu‑CKO^* mice. (**b**) Quantifications of (a). Bar graphs depict the percent change of *Cacna1c/Vglut1*-double positive neurons normalized to controls (*Cav1.2^loxP/loxP^*). (**c**) Selective deletion of *Cacnac1* mRNA in *Vglut2* expressing glutamatergic neurons was assessed by DISH. Deletion of *Cacna1c* expression in *Vglut2* positive neurons was observed in the LDT and MT of *Cav1.2‑Ad^Glu-CKO^* but not in *Cav1.2-Dev^Glu‑CKO^* mice. (**d**) Quantifications of (c). Bar graphs depict the percent change of *Cacna1c/Vglut2*-double positive neurons normalized to controls (*Cav1.2^loxP/loxP^*). Black arrowheads indicate cells only expressing *Cacna1c* (silver grains). Gray arrowheads indicate cells co-expressing *Cacna1c* and the respective neurotransmitter marker (red staining). Red arrowheads indicate cells only expressing the respective neurotransmitter marker. Scale bars: 250 µm (hippocampus overview), 25 µm (higher magnification images). One-Way ANOVA + Bonferroni post hoc test; *p < 0.05, **p < 0.005, *p < 0.0001; n = 4, 1-2 sections/mouse. Data are means ± SEM.


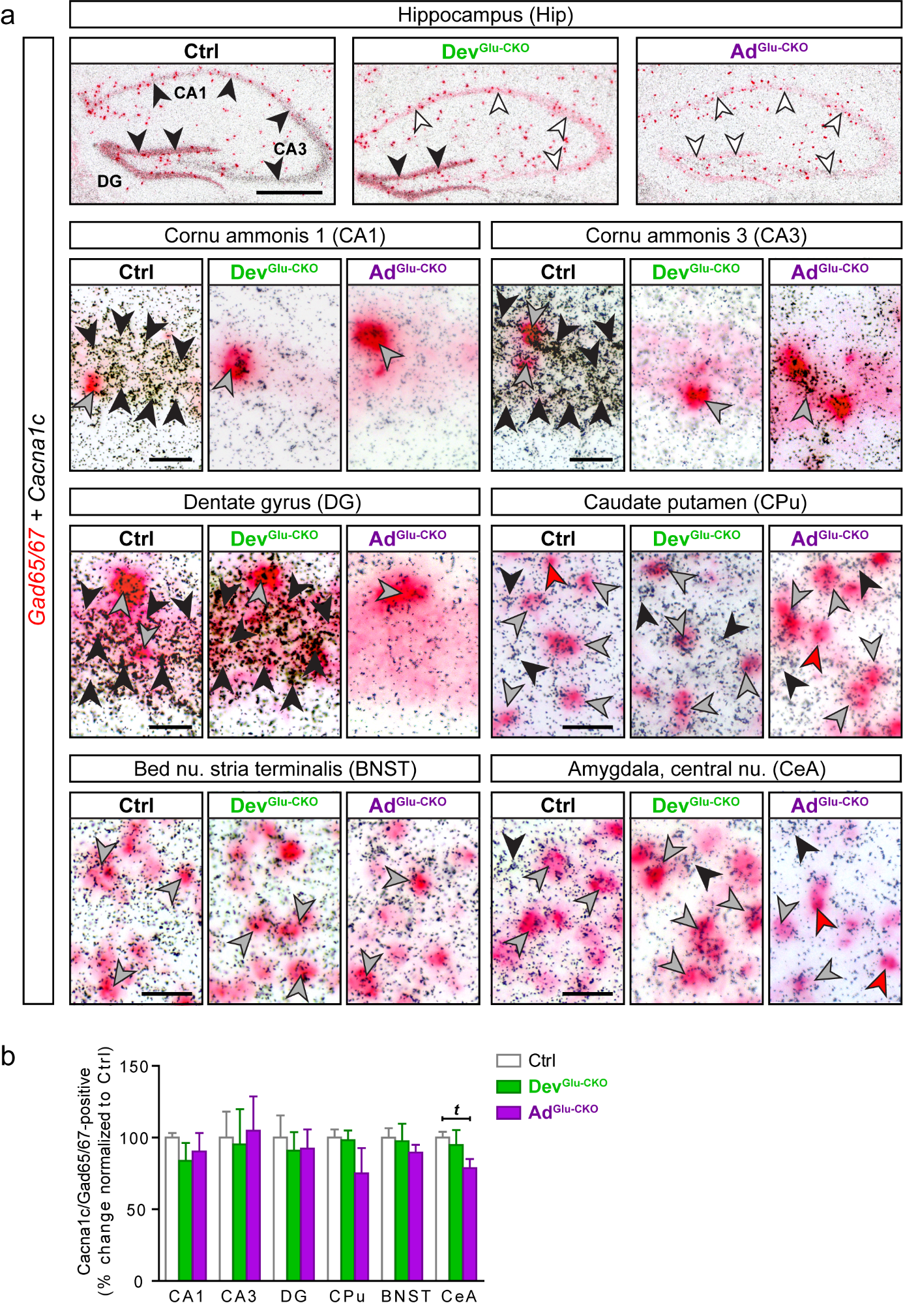


**Figure S4. *Cav1.2-Dev^Glu-CKO^* and *Cav1.2-Ad^Glu-CKO^* mice do not show a prominent loss of *Cacna1c* in GABAergic neurons.**

(**a**) Selective deletion of *Cacnac1* mRNA in GABAergic (*Gad65/67*) neurons was assessed by DISH. No prominent deletion of *Cacnac1* was detected in hippocampal areas CA1, CA3 and DG, as well as the BNST and CPu of *Cav1.2-Dev^Glu-CKO^* and *Cav1.2-Ad^Glu-CKO^* mice. In the CeA, a trend towards a partial *Cacna1c* deletion in GABAergic neurons was detected in *Cav1.2-Ad^Glu-CKO^* but not in *Cav1.2‑Dev^Glu-CKO^* mice. (**b**) Quantifications of (a). Bar graphs depict the percent change of *Cacna1c/Gad65/67*-double positive neurons normalized to controls (*Cav1.2^loxP/loxP^*). Black arrowheads indicate cells only expressing *Cacna1c* (silver grains). Gray arrowheads indicate cells co-expressing *Cacna1c* and Gad65/67 (red staining). Red arrowheads indicate cells only expressing Gad65/67. White arrowheads indicate pyramidal cell layers which lost Cacna1c expression. Scale bars: 250 µm (hippocampus overview), 25 µm (higher magnification images). One-Way ANOVA + Bonferroni post hoc test; *t* = 0.1; n = 4, 1-2 sections/mouse. Data are means ± SEM.


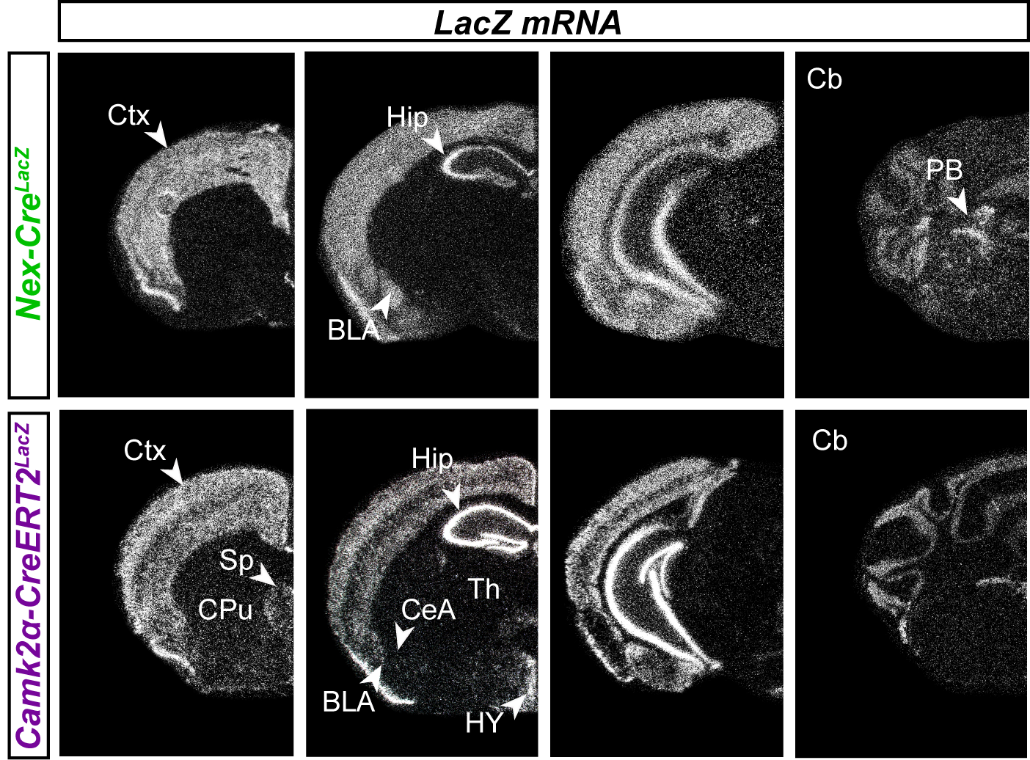


**Figure S5**. **Comparison between *Nex-Cre-* and *Camk2α-CreER^T2^*-driven LacZ mRNA reporter expression patterns.** *Nex-Cre* and *Camk2α-CreER^T2^* mice were bred to Cre-dependent LacZ-Reporter mice resulting in *Nex-Cre^LacZ^* and *Camk2α-CreERT2^LacZ^* mice respectively. *In situ* hybridization revealed a similar *LacZ* mRNA reporter gene expression pattern in both mouse lines. Pronounced LacZ reporter gene expression was detected throughout the cortex and CA1-CA3 of the hippocampus (Hip) of *Nex-Cre^LacZ^* and *Camk2α‑CreERT2^LacZ^* mice. Stronger *LacZ* mRNA expression was detect in the dentate gyrus (DG) of *Camk2α-CreERT2^LacZ^* mice compared to *Nex-Cre^LacZ^* mice. In addition, *Camk2α-CreERT2* activity was also detected in a few cells of the central amygdala (CeA), septum (Sp), caudate putamen (CPu), hypothalamus (HY) and thalamus (Th). *Nex-Cre^LacZ^* driven expression was additionally observed in neurons of the parabrachial nucleus (PB).


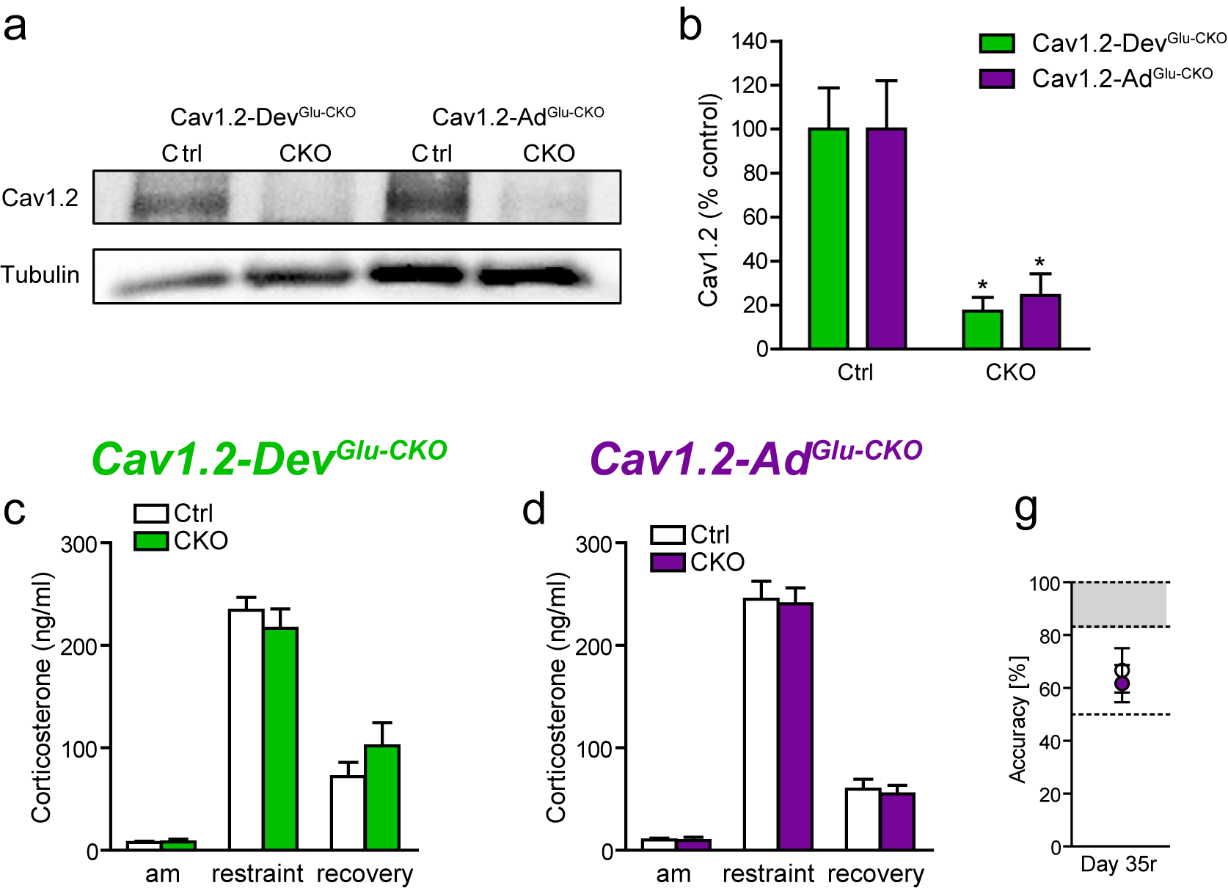


**Figure S6. Additional behavioral and neuroendocrine parameters in *Cav1.2-Dev^Glu-CKO^* and *Cav1.2‑Ad^Glu-CKO^*** **mice.** (**a**) *Cav1.2-Dev^Glu-CKO^* and *Cav1.2-Ad^Glu-CKO^* mice show a similar reduction in hippocampal Ca_v_1.2 protein levels compared to littermate controls, determined by Western blot analysis. (**b**) Bar graphs represent the relative immunoreactivity of Ca_v_1.2 normalized to Ctrl (2-way ANOVA – Mouse line, F_1,10_ = 0.05, p = 0.83; CKO, F_1,10_ = 23.1, p = 0.0007; Bonferroni post-hoc test, p < 0.05; n = 3 per group for *Cav1.2-Dev^Glu-CKO^*, 4 per group for *Cav1.2-Ad^Glu-CKO^*; *significantly different from the control group of the same mouse line). The remaining Ca_v_1.2 immunoreactivity in both mouse lines can be ascribed to Ca_v_1.2-positive GABAergic neurons and in case of *Cav1.2-Dev^Glu-CKO^* mice, also non-recombined cells of the dentate gyrus. (**c,d**) Plasma corticosterone levels during the morning (am), following 10 min of restraint stress (restraint) and after 90 min after restraint stress (recovery) did not differ between genotypes in both *Cav1.2-Dev^Glu-CKO^* (n = 16 Ctrl, 12 CKO) and *Cav1.2-Ad^Glu-CKO^* mice (n = 17 Ctrl, 11 CKO). (**e**) No differences were observed between genotypes in the remote memory trial performed 30 days after the last relearning episode (d35) in the water cross-maze test. *Cav1.2-Dev^Glu-CKO^* mice (n = 11 Ctrl, 10 CKO). Data are means ± SEM.

**
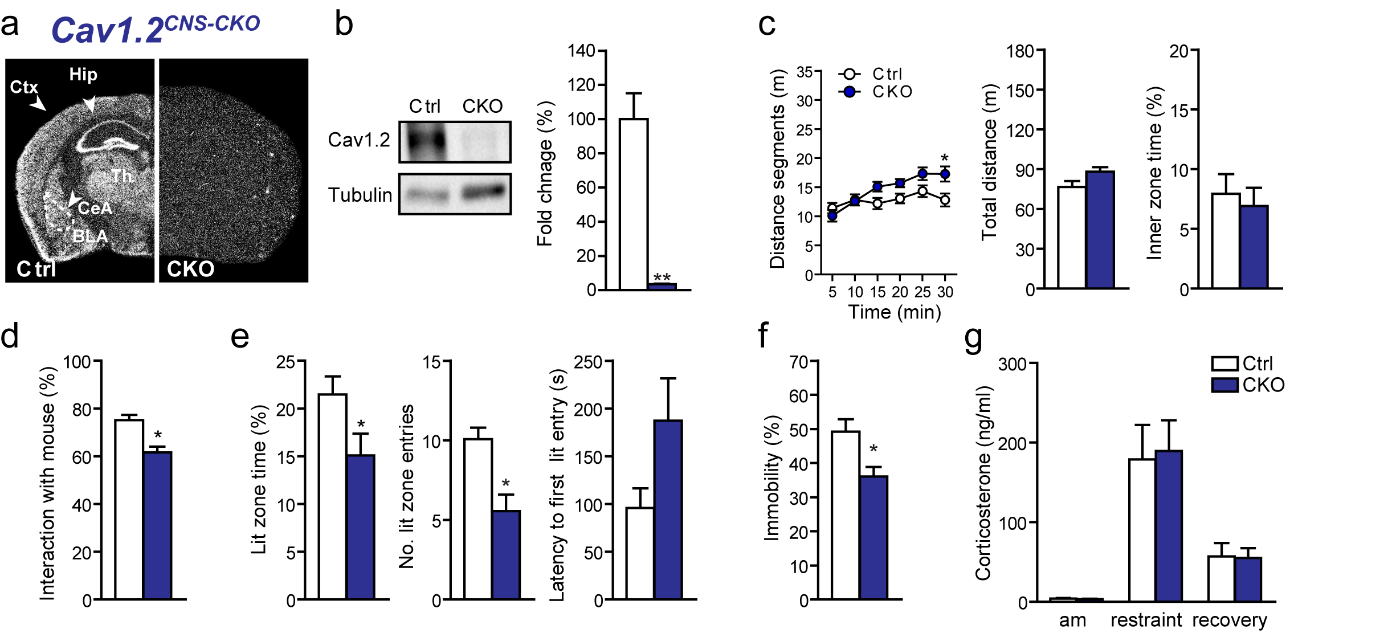
**

**Figure S7.** **CNS-specific deletion of *Cacna1c* results in increased anxiety, hyperactivity and deficits in sociability.** (**a**) *Cacna1c* mRNA expression, determined by ISH, is completely abolished in *Cav1.2^CNS-CKO^* mice. (**b**) Similarly, hippocampal *Cacna1c* protein expression was completely abolished in *Cav1.2^CNS-CKO^* mice. Right: bar graphs represent the relative immunoreactivity of Ca_v_1.2 normalized to Ctrl (Unpaired t-test, t_4_ = 6.4, *p < 0.005; n = 3 per group) (**c**) Deletion of *Cacna1c* from the entire CNS results in hyperlocomotion during the last segments of the OF test (RM-ANOVA – time x genotype: F_5,90_ = 4.1, p = 0.002, Bonferroni post hoc test, *p < 0.05; n = 12 Ctrl, 8 CKO). Total locomotion and inner zone time were not significantly different between genotypes. (**d-f**) Similar to *Cav1.2-Dev^Glu-CKO^* mice*, Cav1.2^CNS-CKO^* mice displayed reduced sociability (Unpaired t-test – t_19_ = 2.8, p = 0.01; n = 13 Ctrl, 8 CKO), increased anxiety in the dark/light box test (Unpaired t-test - Lit zone time (%): t_20_ = 2.2, p = 0.043 / No. lit zone entries: t_20_ = 3.7, p = 0.001; n = 13 Ctrl, 9 CKO) and reduced immobility in the forced swim test (Unpaired t-test – t_20_ = 2.7, p = 0.014; n = 13 Ctrl, 9 CKO). (**g**) Plasma corticosterone levels during the morning (am), following 10 min of restraint stress (restraint) and after 90 min after restraint stress (recovery) did not differ between genotypes (n = 13 Ctrl, 9 CKO). *p < 0.05. Data are means ± SEM. Abbreviations: BLA (basolateral nucleus of the amygdala), CKO (conditional knockout), CeA (central nucleus of the amygdala), Ctx (cortex), Hip (Hippocampus), Th (Thalamus).

**
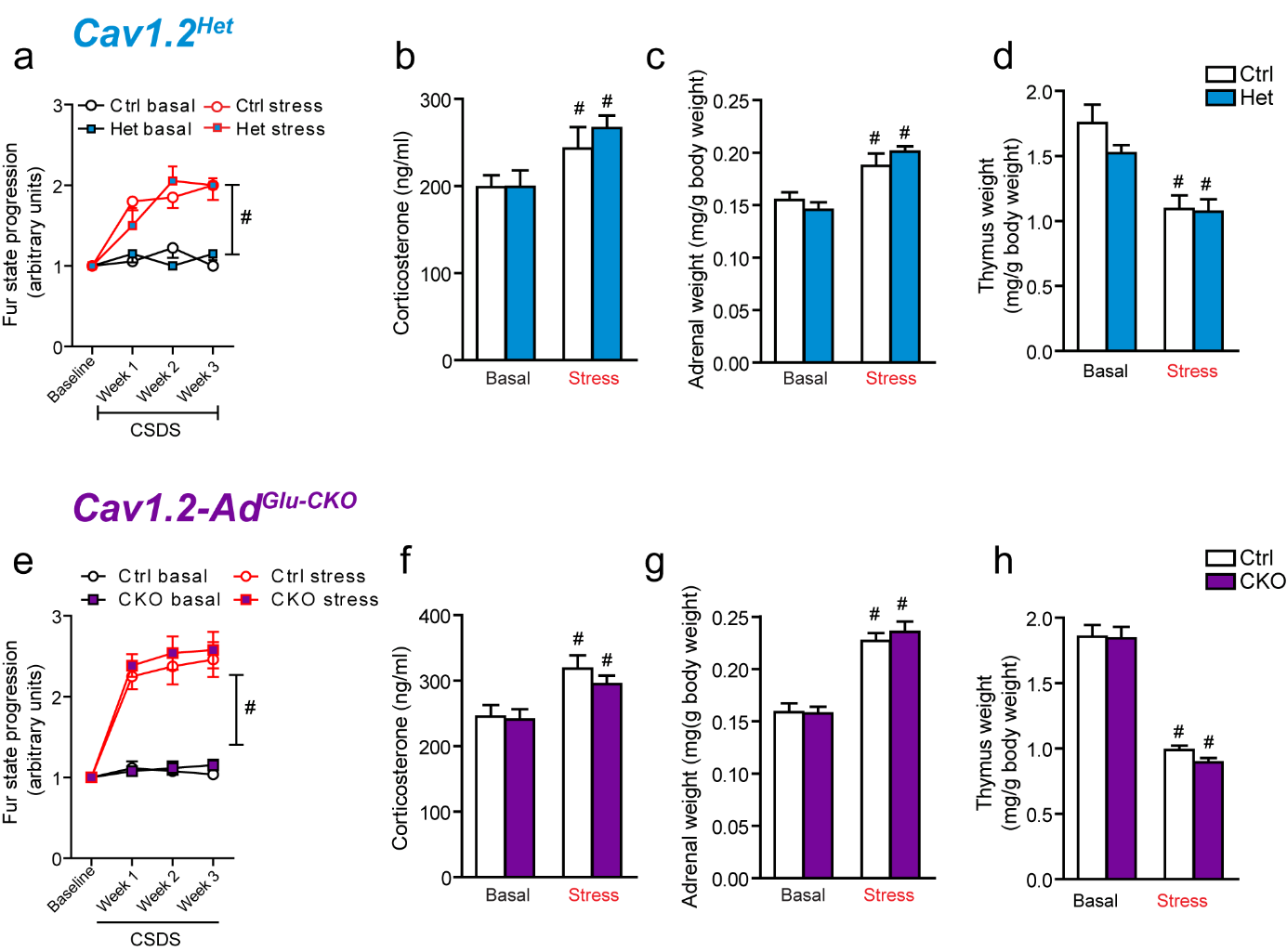
**

**Figure S8. Additional physiological and behavioral parameters in *Cav1.2^Het^* and *Cav1.2Ad^Glu-CKO^* mice following chronic social defeat stress.** (**a**) In *Cav1.2^Het^* mice, CSDS led to a robust decrease in fur state quality, independent of genotype, depicted by a progressive increase in fur coat status (RM-ANOVA - time x stress, F_3,32_ = 28.0, p < 0.0001; stress, F_1,34_ = 75.3, p < 0.0001; n = 9 Ctrl basal, 10 Ctrl stress, 10 Het basal, 9 Het stress). (**b**) Corticosterone response levels (stress, F_1,33_ = 8.54, p = 0.006; n = 9 Ctrl basal, 10 Ctrl stress, 10 Het basal, 8 Het stress) and (**c**) and adrenal gland size (stress, F_1,34_ = 28.2, p < 0.0001; n = 9 Ctrl basal, 10 Ctrl stress, 10 Het basal, 9 Het stress) in *Cav1.2^Het^* mice were increased in both groups following CSDS, which demonstrates the efficacy of the paradigm. (**d**) Similarly, CSDS reduced thymus weight independent of genotype in *Cav1.2^Het^* mice (2-way ANOVA - stress, F_1,34_ = 23.8, p < 0.0001; n = 9 Ctrl basal, 10 Ctrl stress, 10 Het basal, 9 Het stress). (**e)** In *Cav1.2-Ad^Glu-CKO^* mice, CSDS led to a robust decrease in fur state quality in control and CKO mice, depicted by a progressive increase in fur coat status (RM-ANOVA - time x stress, F_3,45_ = 29.0, p < 0.0001; stress, F_1,45_ = 80.2, p < 0.0001; n = 13 Ctrl basal, 12 Ctrl stress, 12 CKO basal, 12 CKO stress). (**f**) Corticosterone response levels (stress, F_1,45_ = 14.9, p = 0.0004) and (**g**) adrenal gland size (stress, F_1,45_ = 79.7, p < 0.0001) were increased independent of genotype (n = 13 Ctrl basal, 12 Ctrl stress, 12 CKO basal, 12 CKO stress). (**h**) Similarly, CSDS induced a significant decrease in thymus weight independent of genotype (2-way ANOVA – stress, F_1,46_ = 187.0, p < 0.0001 (n = 13 Ctrl basal, 12 Ctrl stress, 12 CKO basal, 12 CKO stress). # significantly different from the basal condition of the same genotype, p < 0.05. Data are means ± SEM.

**
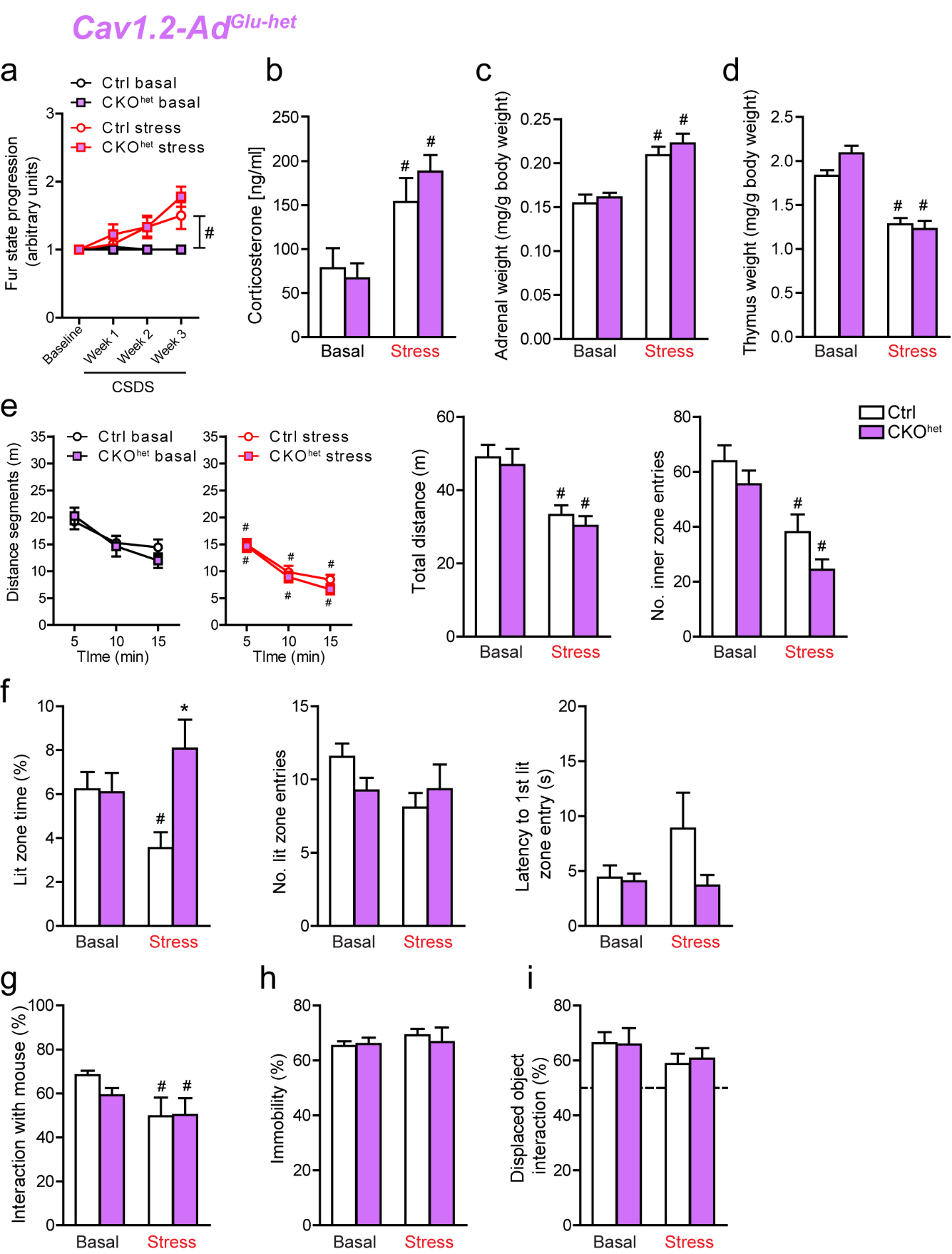
**

**Figure S9. Heterozygous deletion of *Cacna1c* from forebrain glutamatergic neurons during adulthood (*Cav1.2Ad^Glu-Het^*) partially recapitulates resilience to CSDS observed in *Cav1.2Ad^Glu-CKO^* mice.** (**a**) CSDS led to a robust decrease in fur state quality, independent of genotype, depicted by a progressive increase in fur coat status (time x stress, F_3,34_ = 7.95, p < 0.0001; stress, F_1,35_ = 19.85, p < 0.0001). (**b**) Corticosterone levels (stress, F_1,35_ = 17.70, p = 0.0022) and (**c**) and adrenal gland size (stress: F_1,35_ = 37.02, p < 0.0001) were increased in both groups following CSDS, demonstrating the efficacy of the paradigm. (**d**) Similarly, CSDS reduced thymus weight independent of genotype (stress, F_1,35_ = 83.62, p < 0.0001; n = 9). (**e**) Distance traveled and the number of inner zone entries were similarly reduced in Ctrl and CKO mice following CSDS (Distance segments: time, F_2,35_ = 62.13, p < 0.0001; stress, F_2,35_ = 22.96, p < 0.0001 / Total distance: stress, F_1,35_ = 22.96, p < 0.0001 / No. inner zone entries: stress, F_1,35_ = 24.87, p < 0.0001). (**e**) In contrast to Ctrl littermates, the lit zone time was no reduced upon CSDS in CKO mice (stress x genotype, F_1,35_ = 6.20, p < 0.05; genotype, F_1,35_ = 5.52, p < 0.05). No significant effects were observed for the number of lit zone entries and latency to lit zone entry. (**g**) CSDS induced similar deficits in sociability in Ctrl and CKO mice (stress, F_1,35_ = 5.06, p < 0.05). No significant effects were observed in the FST (**h**) and spatial object recognition test (**i**). 2-Way ANOVA + Bonferroni post hoc test, and RM-ANOVA + Bonferroni post hoc test; n = 11 Ctrl basal, 11 Ctrl stress, 8 CKO basal, 9 CKO stress; *significantly different from the control group of the same condition, # significantly different from the basal condition of the same genotype. Data are means ± SEM.

**Table S1**. **SNPs in *CACNA1C* with significant trauma interaction results in the Grady trauma project cohort.**

**Table S2**. **Sample description of the Grady trauma project cohort.**

|  | rs73248708 genotype | | |
| --- | --- | --- | --- |
| mean (sd) | AA | AG | GG |
| age | 45.87 (16.17) | 40.09 (14.08) | 39.97 (13.90) |
| Beck depression index (BDI) | 15.03 (8.03) | 14.32 (12.03) | 14.10 (12.18) |
| Trauma event inventory (TEI) | 5.27 (3.71) | 4.06 (2.91) | 4.10 (2.91) |
| n male/female | 3/4 | 89/242 | 1117/2989 |
|  | rs116625684 genotype | | |
| mean (sd) | CC | CT | TT |
| age | 40.05 (13.96) | 40.14 (13.82) | 39.71 (11.25) |
| Beck depression index (BDI) | 14.23 (12.19) | 13.51 (11.32) | 8.5 (5.47) |
| Trauma event inventory (TEI) | 4.08 (2.92) | 4.22 (3.01) | 4.62 (3.56) |
| n male/female | 1191/3181 | 101/266 | 2/5 |
